# Population-based surveys and interventions for mental health literacy in China during 1997–2018: a scoping review

**DOI:** 10.1186/s12888-019-2307-0

**Published:** 2019-10-26

**Authors:** Shurong Lu, Brian Oldenburg, Wenjing Li, Yanling He, Nicola Reavley

**Affiliations:** 1 Department of Chronic Disease, Jiangsu Provincial Centre for Disease Control and Prevention, Nanjing, 210009 Jiangsu China; 2 The Nossal Institute for Global Health, Melbourne School of Population and Global Health, University of Melbourne, Melbourne, VIC 3010 Australia; 3 Centre for Mental Health, Melbourne School of Population and Global Health, University of Melbourne, Melbourne, VIC 3010 Australia; 4 Shanghai Mental health Centre, Shanghai, 200030 China

**Keywords:** Mental health literacy, Mental disorder, Suicide, Scoping review, China

## Abstract

**Background:**

This scoping review maps population-based surveys and mental health literacy (MHL) interventions undertaken in China during 1997–2018 in order to identify research gaps.

**Method:**

Following Arksey and O’Malley’s framework for a scoping review, five English databases (Medline, PsycINFO, Cochrane library, Web of Science and CINAHL) and two Chinese ones (CNKI and WanFang) were systematically searched, identifying both reports of surveys and evaluation of interventions from Jan 1997 to Oct 2018.

**Results:**

MHL research has developed rapidly in China in terms of numbers of studies and geographic coverage over the past two decades. There were 350 peer-reviewed publications included in this review, covering diverse settings and participants. Of these publications, 313 (89.4%) were published in Chinese-language journals and 37 in English-language journals; 303 (86.6%) reported on survey findings and 47 reported on the evaluation of MHL interventions. MHL research in China has mainly focused on the assessment of mental health-related knowledge and beliefs. Much less attention has been given to developing and evaluating relevant interventions. MHL related to general mental health and suicide were most commonly studied, with less focus on specific disorders, although some studies covered depression, psychosis and anxiety disorders. The majority of MHL tools utilized in the studies reported in this review were developed in China (*n* = 97, 80.2% ) and almost half of these studies (57.8%) did not provide enough details concerning psychometrics.

**Conclusions:**

More interventions targeting the general public and aiming to improve MHL and promote behaviour change, are needed in China. These should be evaluated with high-quality study designs, such as randomised controlled trials. Proper validation of tools used for measuring MHL should also be addressed in future studies.

## Background

Mental, neurological and substance use disorders contribute to a significant proportion of disease burden globally [1]. China accounted for 17% of the global burden of mental, neurological and substance use disorders in 2013 and this is predicted to increase by 10% by 2025 [2]. The latest nationwide epidemiological survey in China reported that the 12-month prevalence of any mental disorder (excluding dementia) among the Chinese population was 9.3% and the prevalence of anxiety, depression and alcohol use disorders in this population all appear to be increasing [3]. Moreover, mental disorders increase the risk of suicide [4, 5] and China has one of the highest suicide rates in the world [6].

In China, the treatment gap for mental disorders is very high [7]. For example, a community-based survey among the general population in a north-western city of China (Xi’an) found that less than one quarter of individuals with a mental disorder diagnosis had sought mental health services in their lifetime [8, 9]. Structural barriers related to mental health service use include a scarcity of available human and financial resources and disparity in their distribution [10]. Individual factors include widespread stigmatising attitudes, poor recognition of mental disorders, low perceived need for treatment and limited knowledge of available services [8, 11]. These individual factors can be summarised by the concept of ‘mental health literacy (MHL)’, which has been defined by Jorm et al. as “knowledge and beliefs about mental disorders that aid in recognition, management, or prevention” [12].

The key components of MHL are: (1) Knowing how to prevent mental disorders; (2) Recognition of when a disorder is developing to facilitate early help-seeking; (3) Knowledge of help-seeking options and available treatments; (4) Knowing effective self-help strategies for milder problems; (5) First-aid skills to support others who are developing a mental disorder or in a mental health crisis [12]. Early surveys in Australia [12] and other high-income countries [13, 14] typically reported relatively low levels of MHL in members of the public, leading to a wide range of interventions and campaigns to improve MHL [15, 16]. Evidence from these interventions has shown that improvements in MHL assisted in promoting early detection of mental disorders, reductions in stigmatising attitudes and enhanced help-seeking behaviours [17, 18].

Since the early 2000s, China’s mental health policies and strategies - notably the *National Mental Health Plans* released in 2002 and 2015 - have aimed to promote the development of the Chinese mental health system with respect to narrowing the treatment gap [19]. Each National Plan set specific goals relating to the awareness rate of mental health related knowledge among urban and rural residents [20], leading to an increase in population-based surveys and interventions for mental health related knowledge, attitudes and skills [21–23].

MHL is a relatively new term in the Chinese language [24]. In 2007, an epidemiological survey conducted in three cities of China (Beijing, Shanghai and Changsha) first employed this term to estimate recognition rates and attitudes to mental illness [25]; and He and Wang introduced the concept of MHL in a Chinese journal in 2013 [26]. However, some components of MHL, predominantly related to attitudes and knowledge, have been studied in China for many years [27]. For example, a variety of research activities on stigmatising attitudes towards mental illness had been conducted among the Chinese population [11, 28]. Examples of culturally specific beliefs about the causes of mental health problems in traditional Chinese culture include viewing mental illness as a punishment for the misconduct of family members and/or their ancestors, or beliefs that mental disorder are inflicted by a supreme being [29]. To further explore and counter these beliefs and to disseminate scientific knowledge, a number of studies focusing on knowledge about mental health problems have been conducted in China [22, 23, 30].

Similarly, a variety of studies have assessed suicide-related knowledge and attitudes in Chinese culture [31]. It has been argued that some of the characteristics of suicides in China [32] (e.g. a 1.0:1.3 male to female ratio compared with the approximately 3:1 male–female ratio observed in Western countries) are related to Chinese Confucian culture (encouraging suicide for the pursuit of ‘loyalty (*Yi* or *Ren,* in the Chinese language)’) and Buddhist beliefs (reincarnation) [33].

Despite the increasing focus on MHL research in China, we lack a comprehensive overview of the studies that have been conducted in this field. In order to develop specific strategies to achieve the goal of improving population MHL, it is necessary to understand the research and program activities that are currently being conducted so as to identify key research gaps. Therefore, we conducted this scoping review in order to identify study types, geographic locations, study settings, domains of MHL, MHL tools and characteristics of existing interventions, and to identify gaps and consider implications for future research.

## Methods

A scoping review describes and summarizes the literature in an area of interest. It aims to map the relevant literature and studies in the field of interest, rather than focusing on a specific research question. It can also highlight gaps in the evidence base. Unlike a traditional systematic review, it can incorporate studies using different methodologies [34] and may provide the foundation for a more specific systematic review and meta-analysis in the future. For this scoping review, we adopted the methodological framework of Arksey and O’Malley [35].

To incorporate key components of MHL and to facilitate data extraction, we categorised MHL into the following seven domains: (1) General knowledge of mental health/disorder(s); (2) Symptom recognition; (3) Knowledge/beliefs about causes or risk factors; (4) Knowledge/beliefs about treatment/help-seeking (including intentions and behaviours); (5) Beliefs about self-help strategies; (6) Beliefs about helping others/first aid, and (7) Other components which could not be categorized into any of above initial domains.

### Search strategy

Five English databases (i.e. Medline, PsycINFO, Cochrane library, Web of Science and CINAHL) and two Chinese ones (CNKI and WanFang) were systematically searched for peer-reviewed publications reporting on population-based surveys and/or evaluation of interventions in the field of MHL in mainland China. Search terms consisted of three concepts, i.e. mental disorders, domains of MHL and study regions, linked by a Boolean Operator of “AND”. Among each of these concepts, general terms like “mental disorders”, “mental health literacy”, “China” and individual common phases such as “depression, anxiety, schizophrenia or suicide”, “health knowledge, attitudes, practice, help-seeking behaviour” “Chinese people, Hong Kong, Taiwan, Macau”, or their synonyms were used for search. These terms were used in varying combinations to identify relevant literature in different databases (see online Additional file 1 for detailed search strategy and search terms). Since the concept of MHL was introduced and defined by Jorm et al. in the year of 1997 [12], studies conducted before 1997 were excluded. Searches were carried out between Sep 2017 and Mar 2018 and updated in Nov 2018.

### Inclusion and exclusion criteria

We included reports of observational surveys or experimental interventions with primary quantitative data on one or more of the seven domains of MHL as defined above. Given the differences between health systems in mainland China and Hongkong, Taiwan and Macau, we excluded studies in these regions. Since mental illness-related stigmatising attitudes in China have been previously reviewed [22, 23, 28], studies focused only on stigma or attitudes towards people with mental disorders were also excluded. In cases where more than one paper reported data from the same study and the information was repeated, the study with relatively greater information was retained in the scoping review. We also excluded studies that specifically assessed changes in knowledge due to clinical training in mental health professionals, although studies that aimed to improved MHL in other health professionals were included.

### Study selection

**Fig. 1:**
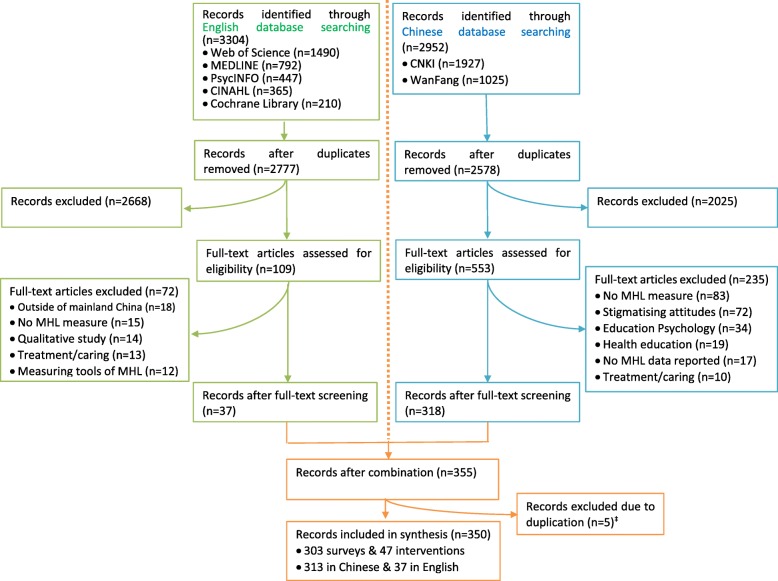
Flow diagram of study selection for the scoping review of MHL in China, 1997–2018^†^. ^†^The year of 2018 includes the first 10-month only; ^‡^Five Chinese papers excluded due to duplication with English ones with more detailed information

Endnote software (Version X8) was used to help with literature screening. Literature selection was performed in accordance with the PRISMA-ScR checklist [36]. Duplicates were firstly removed by using the ‘find duplicates’ function in EndNote and hand checking. Irrelevant studies were then excluded by reviewing titles and abstracts. Full texts of relevant studies identified from the title and abstract screen were obtained and subsequently assessed for eligibility (by author SL, with independent screening of the English articles by author NR and the Chinese ones by author WL). The reference lists of relevant reviews were also checked for additional studies. The review process for the literature in Chinese and English languages is outlined separately in Fig. 1.

### Data extraction and synthesis

Two data extraction forms (one for surveys and one for interventions, see online Additional file 2) were firstly developed based on the framework proposed by Jorm et al. [12] and modified according to findings from pilot testing on 50 included studies, which included the Mental Health Knowledge Questionnaire (MHKQ) – an instrument developed specifically for China. Variables of interest included basic information (first author, year of publication, study setting), study design, type of participants, recruitment method, sample information (varied between surveys and interventions), MHL tools (referring to questionnaires or scales used to measure any domain of MHL) and their validation, mental disorder targeted, and domains of MHL measured. For the latter, we extracted relevant information from each publication (as described in each full-text article or based on the contents of the relevant MHL tool) into a separate document, which was then imported into NVivo software (Version 12) for thematic analysis. Initial thematic analysis used the framework outlined by Jorm et al. [12], which was then modified according to the new themes that emerged. As the MHL domains vary among different types of participants, MHL domains were firstly analysed according to the type of participant (i.e. lay people, professionals and patients/carers), followed by three broad categories of MHL (i.e. knowledge, beliefs and behaviours/skills), and then more detailed subthemes.

In the extraction form for interventions, intervention information (e.g. case/control information, approach, delivery of the intervention) was also included. Data were extracted independently by authors SL and WL and finalised after cross-checking and discussion with author NR. Characteristics of included studies/publications were reported after synthesis. Frequencies and percentages were used to examine the distribution of included studies/publications.

## Results

### General characteristics of included studies

**Fig. 2:**
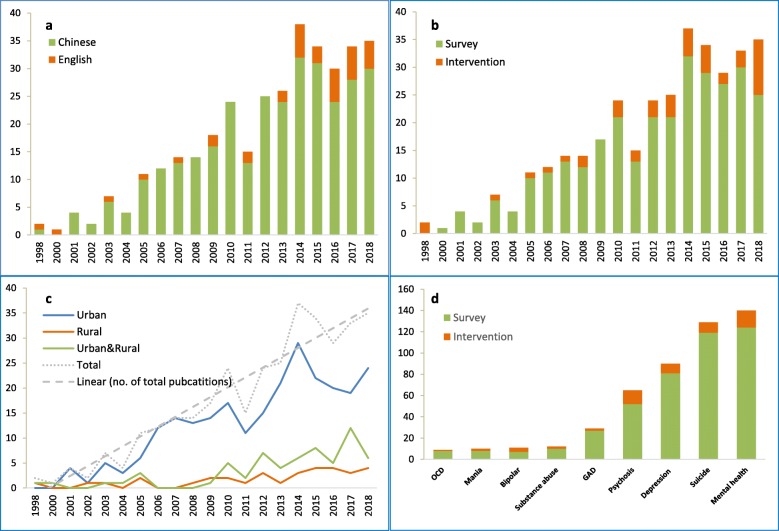
Characteristics of included literature: **a** Language by year†; **b** Study type by year; **c** Study area by year; **d** Study type by measured disorder‡ (with a total number of 350 publications included). ^†^The year 2018 includes the first 10 months only and there were no studies identified for the years 1997 and 1999; ^‡^Some studies covered multiple disorders; OCD: obsessive-compulsive disorder; GAD: generalised anxiety disorder

A total number of 350 articles were included in this review (see online Additional file 3 for the full list of included articles). Among these articles, 313 were published in Chinese language and 37 were in English; 303 reported descriptive data from surveys and 47 reported on the evaluation of interventions. An increase in the number of MHL-related publications during 1997–2018 in China was seen, growing from 0 in 1997 to 35 in 2018 (Fig. 2a). The number of papers on interventions for MHL has also increased, particularly since 2014 (*n* = 11, Fig. 2b).

However, as illustrated in Fig. 2c, this increase was largely due to a rise in the number of studies in urban areas, whilst there was only a slight increase in studies in rural areas (from 1 in 1998 to 4 in 2018). Overall, many more MHL studies were conducted in urban than rural areas of China in the past two decades (251 vs. 32). Notably, the number of studies involving both urban and rural areas (*n* = 67) has steadily increased since 2010. Most of these were epidemiological surveys.

### Mental disorder focus

In terms of mental disorder focus, most studies (*n* = 140, including 124 surveys and 16 interventions) assessed MHL of mental health broadly rather than focusing on specific disorders. The next most common topic was suicide (*n* = 129, including 119 surveys and 10 interventions). Depression, psychosis and generalised anxiety disorder (GAD) were the top three disorders for which MHL was assessed (*n* = 90 for depression, 65 for psychosis and 29 for GAD, respectively). However, other common mental disorders, such as substance use disorders, bipolar disorder, mania or obsessive-compulsive disorder (OCD) have been less commonly studied. No studies on MHL related to trauma or post-traumatic stress disorder (PTSD) were identified, suggesting a gap for future research (Fig. 2d).

### Geographic distribution

**Fig. 3:**
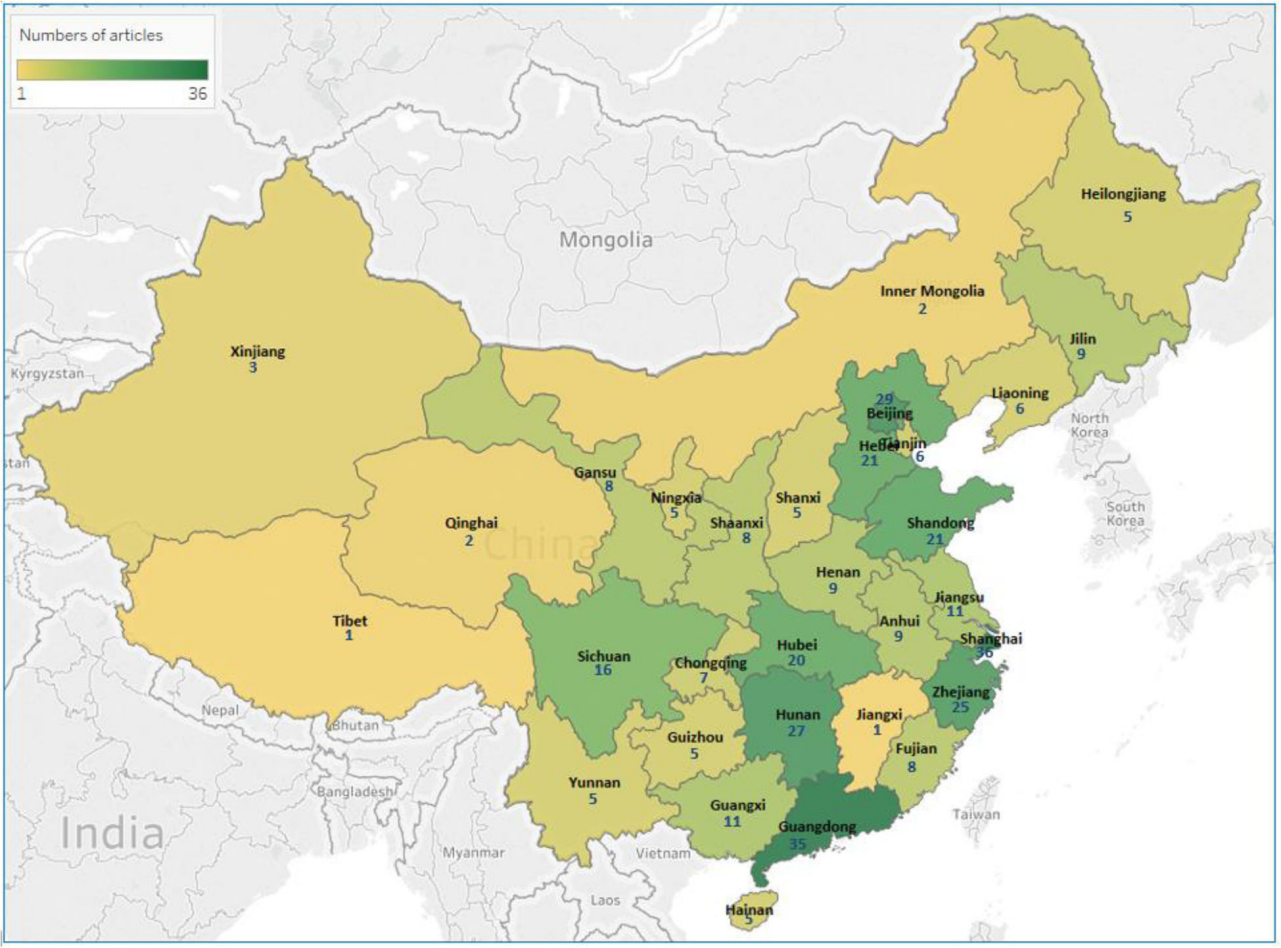
Number of MHL publications in the 31 provincial regions of mainland China, 1997–2018^†^. ^†^The year of 2018 includes the first 10-month only; There were 3 nationwide studies not shown, 8 studies across 2 provinces and 3 studies across 3 provinces; This map was generated using the Tableau software (Version 10.4, product key: TCXX-1320-B080-7BE1-818F)

The 350 MHL-related publications during 1997–2018 covered all the 31 provincial regions of mainland China, but they have been distributed unevenly across the country. Provinces with most publications are Shanghai (*n* = 36), Guangdong (*n* = 35), Beijing (*n* = 29), Hunan (*n* = 27), Zhejiang (*n* = 25), Shandong and Hebei (*n* = 21, respectively) and Hubei (*n* = 20). These eight provinces accounted for nearly 60% of all MHL-related publications in China in the past two decades (*n* = 214, out of 350), although only about 30% of the Chinese population lives in these provinces (according to data from the latest Chinese population census [37]). By contrast, the 12 provinces in the very north or west of China, such as Heilongjiang, Inner Mongolia and Yunnan, had just 5 or fewer publications in this field during the same period (the total number of MHL publications in the 12 provinces was 44) (Fig. 3).

### Study settings, participants, and recruitment methods

**Fig. 4:**
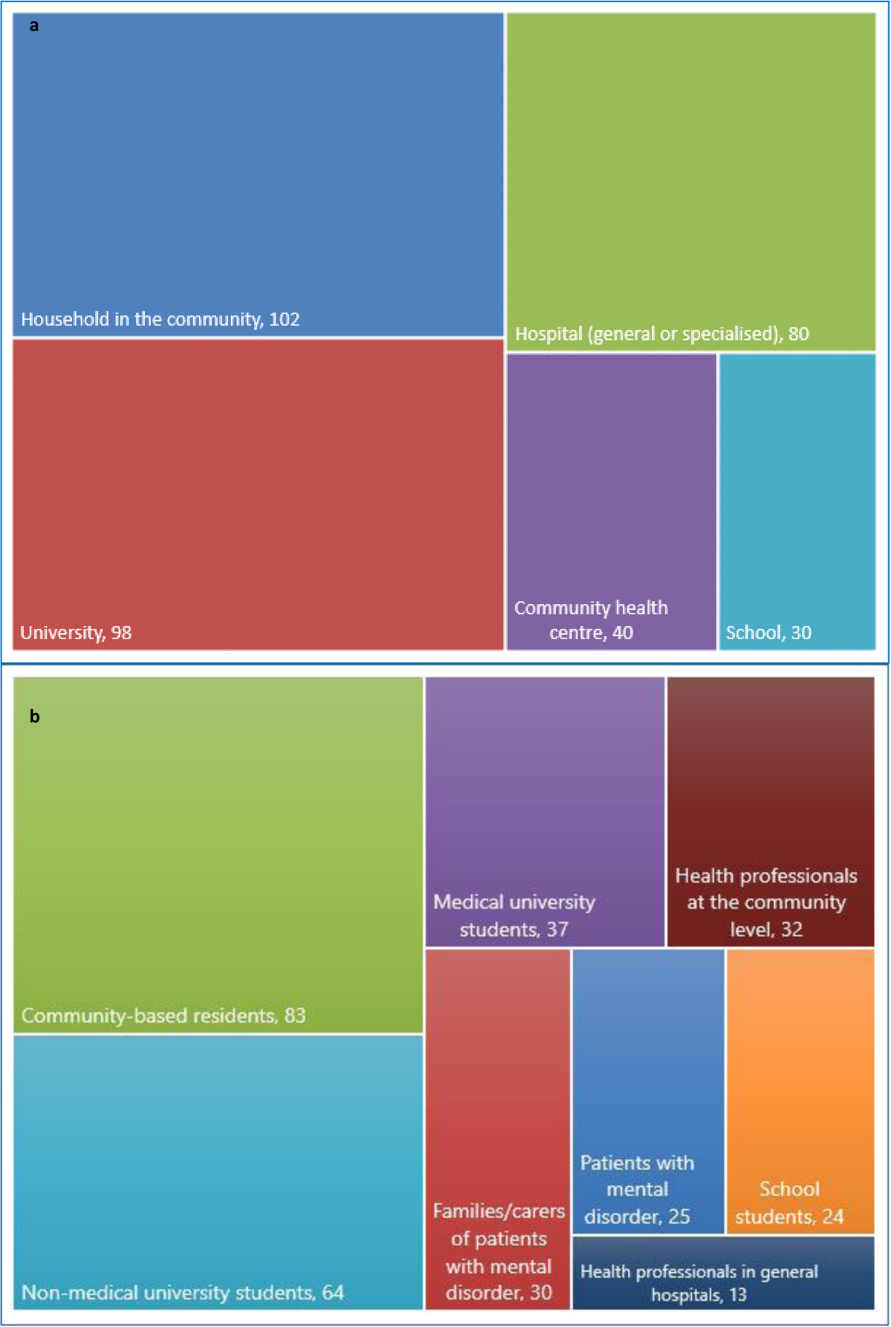
Tree-map charts of **a**, Study settings and **b**, Participants^†^ (with a total number of 350 publications included). ^†^Some studies covered multiple settings or several types of participants; categories with frequency < 10 were not shown

As shown in Fig. 4a, households in the community, universities, hospitals, community health centres and schools (primary or secondary) were the most common settings for MHL studies in China during 1997–2018, although this differed between surveys and interventions. Specifically, surveys were more likely to happen in the community (30.1%) or universities (29.8%), whereas interventions were predominantly conducted in hospitals (40.5%) or community health centres (27.0%). Accordingly, community-based residents and university students (medical or non-medical) were the most common participants in MHL surveys, while patients with mental disorders, carers and health professionals were more common participants in MHL-related interventions. Workplaces, mostly civil services, were mentioned as study settings in a few surveys, but not in any interventions.

Taking surveys and interventions together, the most common participants in MHL research in China were university students (*n* = 101, of which 64 were non-medical and 37 medical), community-based residents (*n* = 83), health professionals at the community level (i.e. GPs, *n* = 32) and carers of patients with mental disorders (*n* = 30), followed by patients with mental disorders (*n* = 25), primary/secondary school students (*n* = 24) and health professionals in general hospitals (*n* = 13) (Fig. 4b).

### MHL domains and their combination patterns

**Fig. 5:**
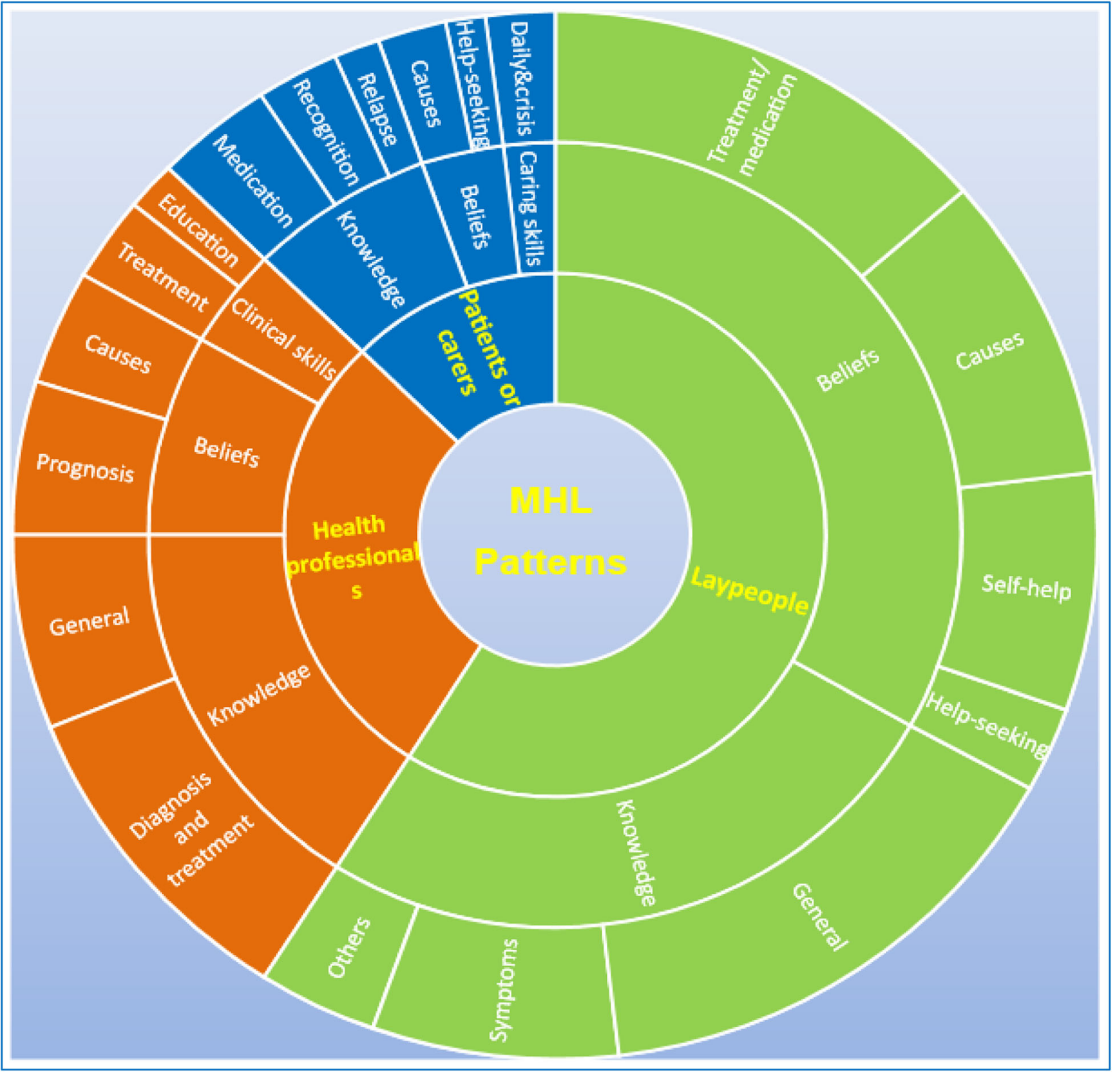
Sunburst graph of MHL domain combination patterns^†^ (with a total number of 350 publications included). ^†^Patterns were identified by the similarity of MHL domain combination among different groups of participants. The ‘lay people’ pattern refers to MHL domains mostly reported in studies of community-based residents, students (university or school), and others; the ‘health professionals’ pattern refers to MHL domains mostly reported in studies among health professionals (specialised, general, or GPs); and the ‘patients or carers’ pattern refers to MHL domains studied among patients with mental disorders and their carers

Figure 5 demonstrates the frequency of different MHL domains assessed by study population. For lay people (community residents, university students and other groups of lay people), knowledge and beliefs were the two most highly examined components. Assessment of knowledge was mostly targeted to mental health broadly, rather than to specific disorders. The emphasis was on knowledge of typical symptoms and recognition of common mental disorders, as well as health service availability, the mental health law or legal rights of patients. Questions about treatment beliefs mainly involved the effectiveness of professional treatments/antipsychotic medication, self-help and help-seeking intentions. Other questions covered causes and risk factors.

In addition to assessment about knowledge and beliefs, assessment of MHL in health professionals and patients or carers covered specific skills. These skills included clinical skills related to the provision of treatment and health services for non-mental health professionals in hospitals, skills to follow-up patients with mental illness in the community for GPs, caring or crisis management skills for patients and their carers (Fig. 5).

General knowledge about mental health was also one of the key domains assessed in health professionals. While knowledge about how to make diagnoses and provide treatment was the key construct assessed for health professionals (i.e. specialised, general, or GPs), for patients and carers, knowledge was mostly about medication (e.g. common medications for specific mental illness, compliance with antipsychotic drug regimens) and how to recognise symptoms of relapse or how to prevent the occurrence of relapse. Regarding beliefs, prognosis was assessed in health professionals and help-seeking preference was assessed in patients and carers. Assessment of beliefs about causes was seen in all groups (Fig. 5).

### Features of interventions

While an overwhelming majority of MHL research to date in China has focused on surveys rather than interventions (303 vs. 47), more than 60% (*n* = 31) of these interventions used a Knowledge, Attitude, and Practice (KAP) approach, which is common in health education. Delivery methods included face-to-face lectures, bulletins or booklets, although a few MHL studies used computers [23, 38] or the internet [39]. Related to the commonly-used KAP delivery method, most interventions focused on evaluation of changes in knowledge or attitudes. Though a limited number of surveys asked about respondents’ intentions to provide help or first aid to others with mental health problems, no interventions aiming to promote such actions were found in this review. Furthermore, compared to the relatively large number of interventions targeted to patients or their carers (*n* = 21, accounting for 44.7% of all interventions) and non-mental health professionals and GPs (*n* = 16, 34.0%), little attention has been paid to MHL interventions for members of the public (*n* = 10, 21.3%).

### MHL tools and their validation

A total number of 121 MHL tools were reported in the 350 papers of this review, of which 51 were previously validated (27 of them were developed in China and 24 adapted from overseas) and 70 were self-designed for individual studies. The percentage of publications using self-designed tools were 30% during 1997–2009 and 16% during 2010–2018. The MHL assessment tools developed by the Chinese Ministry of Health in 2010 [40] and the Shanghai Mental Health Centre in 2005 [41] were the most widely adopted measures of MHL in China during 1997–2018. No psychometric information about the tools developed by the Chinese Ministry of Health (2010) was found, except a validation study of one (out of eight) of its questionnaires about mental health knowledge awareness among middle school students [42], despite this questionnaire being originally developed for the general population. The tool developed by the Shanghai Mental Health Centre has been partly validated [43]. The majority of the studies describing the 70 self-designed MHL tools identified in this review (accounting for 57.9% of all tools included) included little or no psychometric information.

The Questionnaire on Suicide Attitudes (QSA) [44] and the Scale of Public Attitudes about Suicide (SPAS) [45] have been mostly used to measure knowledge of or attitudes to suicide. These tools have been shown to have good validity and reliability among the Chinese population [46, 47].

## Discussion

This scoping review mapped the peer-reviewed literature relating to MHL surveys and interventions to improve MHL in China during 1997–2018. It included 303 epidemiological surveys and 47 interventions and described their geographic distribution, study design, mental disorders, assessment tools and domains of MHL covered.

### Geographic distribution of studies

This review demonstrates a significant increase in MHL-related publications in China over the past two decades. However, there are differences in their geographic distribution, with most studies focusing on urban areas (*n* = 251, 71.7%) (see Fig. 2c) and provinces in the east or south of China (see Fig. 3). Such geographic differences may be related to the uneven distribution of mental health service resources, which are clustered in and around big cities in economically booming areas in China [19]. The lack of locally available mental health service networks and mental health resources make it challenging to conduct MHL-related research in rural, remote or other less-developed regions. On the other hand, due to relatively low levels of education and the strong influence of traditional culture, people in these regions are more likely to have poorer MHL [48], longer delays in receiving treatment, and more premature death related to mental disorders [49]. Therefore, more high-quality MHL research in rural and less-developed regions in China is needed, and could be considered a matter of priority for the Central and local governments of China to achieve the aspirational goal of knowledge awareness rates set by its *National Mental Health Plan* [20].

### Disorders studied

The largest number of studies included in the review involved assessment of MHL related to general mental health and suicide, followed by disorders of depression, psychosis and anxiety. In countries such as Australia where there has been a substantial increase in MHL research over the past 10 years, there has been a major focus on common mental disorders, most notably depression [17, 18]. Given the importance of early help-seeking, self-help and social support in reducing the burden of disease related to depression, this may also be a useful strategy for public education in China. The increasing prevalence of depression [50] and more mass media reports of depression cases or suicide cases related to depression among celebrities may also contribute to the greater awareness of depression among the population [51]. Psychosis has long been a priority in mental health management in China, partly due to its relatively greater burden on individuals and families, as well as its perceived influence on the safety and stability of the whole society [10]. The relatively high number of studies focusing on suicide is likely to be explained by concern about the suicide rate in China, which is among the highest in the world. In comparison, anxiety disorders have been the focus of relatively little MHL research (*n* = 29), although these disorders are reported to be the second most prevalent category of mental disorders in China [9] (with the first being mood disorders). The limited number of studies on substance use, bipolar disorder, mania or OCD and the gap in trauma/PTSD studies highlight the need for a wider range of mental disorders to be covered in the field of MHL research.

### Need for more intervention research

Some studies have shown that Chinese people are more apt to seek help from family and friends to deal with mental health problems before seeking help from a health professional [24]. If a member of the public would like to provide such help, they need to have adequate knowledge, positive attitudes and relevant skills. However, this review suggests that current MHL interventions in China have paid relatively little attention to members of the public but have focused mainly on patients/their carers or non-mental health professionals. While there is a need to further support carers of people with severe mental illness, it is also necessary to improve MHL in members of the Chinese public to enable them to better assist those with mental health problems in their social networks. This is in line with the aims of the World Health Organisation and the Chinese government to improve mental health of the whole population [20, 52].

Moreover, mental disorders such as anxiety and depression are common (the 12-month prevalence among Chinese population is 5.0% for anxiety and 3.6% for depression) [3], many people will have contact with someone with one of these mental disorders. As delays in recognition and help-seeking lead to poor prognosis, empowering friends and family to recognise the signs and symptoms of a mental disorder, encourage a person to seek help and provide support even if they are not in a carer role, may assist in reducing the burden of disease related to these disorders.

It seems likely that MHL among the general population in China will improve due to a greater focus on measurement of it and interventions to improve it and it is hoped that the findings of this review may be useful for other lower resource settings as they undertake mental health reform. For example, a relatively large number of studies targeted university students, which might be explained by a policy focus on mental health in educational institutions and the fact that universities are a relatively convenient setting in which to target young people (the age at which many mental health problems have their first onset). This lays a foundation for developing interventions to assist members of the public to help others with mental health problems in the community. As the Chinese mental health system faces considerable challenges due to the increasing numbers of people seeking help for mental disorders [3, 9] and the severe shortage of mental health professionals [48], such interventions could play a role in building capacity in non-mental health professionals and empower community members to work together to improve population mental health [7].

### Measuring tools and their psychometric properties

Valid measurement tools are important for assessment of MHL and Chinese researchers and relevant government departments have been working on development of MHL tools which are culturally appropriate for Chinese populations. As shown in this review, diverse tools have been used in the field of MHL research in China in recent decades. Some of these are of good quality and have been widely adopted in relevant research [43, 46, 47], which facilities comparison between studies. Still, insufficient validation of tools used for measurement of MHL found in this review should be addressed in future studies.

### Strengths and limitations

A key strength of this review is its coverage of Chinese- and English-language literature. Nevertheless, it is important to consider several limitations of this review when interpreting its findings. While this review was comprehensive and broad, we did not perform any quality assessment of included publications, although we only included peer-reviewed publications. MHL related studies published before the year of 1997 were not included, but we believe that this number is not large judging by the time trend of MHL publications observed in this review.

## Conclusions

Research in population-based surveys and interventions to improve MHL in China has developed quite rapidly over the last 20 years in term of numbers of studies and geographic coverage. The research has involved a diversity of settings and participants. MHL research into a broader range of mental disorders, such as substance use disorders, bipolar disorder and trauma, are warranted. Interventions targeted to the general public and aiming to improve MHL and promote behaviour change, including help seeking and skills to help people who are developing mental health problems, are also needed. Such programs should be evaluated with higherquality study designs, such as controlled trials. Proper validation of tools used for MHL measurements should also be addressed in future studies. Findings of this review provides evidence to policy makers, practitioners and consumers, and assists in underpinning future research areas.

## Supplementary information

**Additional file 1.** Searching strategy.

**Additional file 2.** Data extraction forms.

**Additional file 3.** List of included papers.

## Data Availability

Data used for this scoping review is a number of 350 publications, and a full list of these publications is presented within the Additional file 3 of this paper.

## Abbreviations

GAD: Generalised anxiety disorder
KAP: Knowledge, Attitude, and Practice
MHL: Mental health literacy
OCD: Obsessive-compulsive disorder
PTSD: Post-traumatic stress disorder
QAS: The Questionnaire on Suicide Attitudes
SARS: Severe acute respiratory syndrome
SPAS: The Scale of Public Attitudes about Suicide
