# Supplementary material for: Population-based surveys and interventions for mental health literacy in China during 1997–2018: a scoping review
Source: BMC Psychiatry. 2019 Oct 26;19:316. doi: 10.1186/s12888-019-2307-0 (PMC6815452; doi:10.1186/s12888-019-2307-0)
Supplement: Supplementary file 3 — Additional file 3. List of included papers. [file 12888_2019_2307_MOESM3_ESM.docx]

1. Furnham A, Wong L: A cross-cultural comparison of British and Chinese beliefs about the causes, behaviour manifestations and treatment of schizophrenia. Psychiatry Research 2007, 151(1-2):123-138.
2. Cui. G, Li. H, Yao. M: A mental health training programme for Chinese rural general practitioners. Med Educ 2015, 49(5):538-540.
3. Liu W, Gerdtz M, Liu T: A survey of psychiatrists' and registered nurses' levels of mental health literacy in a Chinese general hospital. International Nursing Review 2011, 58(3):361-369.
4. Chen H, Wang Z, Phillips MR: Assessing knowledge and attitudes about mental illness in Ningxia, China. Transcult Psychiatry 2017:1363461517748847.
5. Zhang J, Jia CX: Attitudes toward Suicide: The Effect of Suicide Death in the Family. Omega-Journal of Death and Dying 2009, 60(4):365-382.
6. Zou YM, Leung R, Lin S, Yang MA, Lu T, Li XY, Gu J, Hao C, Dong GH, Hao YT: Attitudes towards suicide in urban and rural China: a population based, cross-sectional study. Bmc Psychiatry 2016, 16.
7. Lu Y, Arthur D, Hu LL, Cheng G, An FR, Li Z: Beliefs about antidepressant medication and associated adherence among older Chinese patients with major depression: A cross-sectional survey. International Journal of Mental Health Nursing 2016, 25(1):71-79.
8. Liu W, Li Y-M, Peng Y: Beliefs about prognosis and outcomes for people with mental disorders: A cross-cultural study of Bachelor of Nursing students from the US and China. Archives of Psychiatric Nursing 2018, 32(5):751-756.
9. Phillips MR, Li Y, Stroup TS, Xin L: Causes of schizophrenia reported by patients' family members in China. Br J Psychiatry 2000, 177:20-25.
10. Wong DFK, Cheng C-W, Zhuang XY, Ng TK, Pan S-M, He X, Poon A: Comparing the mental health literacy of Chinese people in Australia, China, Hong Kong and Taiwan: Implications for mental health promotion. Psychiatry Research 2017, 256:258-266.
11. Stefanovics E, He H, Ofori-Atta A, Cavalcanti MT, Rocha Neto H, Makanjuola V, Ighodaro A, Leddy M, Rosenheck R: Cross-National Analysis of Beliefs and Attitude Toward Mental Illness Among Medical Professionals From Five Countries. Psychiatric Quarterly 2016, 87(1):63-73.
12. Eskin M, Kujan O, Voracek M, Shaheen A, Carta MG, Sun JM, Flood C, Poyrazli S, Janghorbani M, Yoshimasu K et al: Cross-national comparisons of attitudes towards suicide and suicidal persons in university students from 12 countries. Scandinavian Journal of Psychology 2016, 57(6):554-563.
13. Jiao YM, Phillips MR, Sheng YR, Wu GJ, Li XY, Xiong W, Wang LW: Cross-sectional study of attitudes about suicide among psychiatrists in Shanghai. Bmc Psychiatry 2014, 14.
14. Wong DFK, Li JCM: Cultural Influence on Shanghai Chinese People's Help-Seeking for Mental Health Problems: Implications for Social Work Practice. British Journal of Social Work 2014, 44(4):868-885.
15. Ran MS, Xiang MZ, Chan CLW, Leff J, Simpson P, Huang MS, Shan YH, Li SG: Effectiveness of psychoeducational intervention for rural Chinese families experiencing schizophrenia - A randomised controlled trial. Social Psychiatry and Psychiatric Epidemiology 2003, 38(2):69-75.
16. Li Z, Arthur D: Family education for people with schizophrenia in Beijing, China: randomised controlled trial. In: British journal of psychiatry. vol. 187; 2005: 339-345.
17. Xiaolu R, Wenwen W, Ali R, Xu L, Hong W, Min Z, Jiang D: Feasibility of Studying a Brief Intervention to Help Chinese Villagers with Problem Alcohol Use After an Earthquake. Alcohol & Alcoholism Supplement 2017, 52(4):472-476.
18. Zhou JS, Xiang YT, Li QG, Zhu XM, Li W, Ungvari GS, Ng CH, Ongur D, Wang XP: Gender differences in attitudes towards antipsychotic medications in patients with schizophrenia. Psychiatry Research 2016, 245:276-281.
19. Zhang M, He Y, Gittelman M, Wong Z, Yan H: Group psychoeducation of relatives of schizophrenic patients: two-year experiences. Psychiatry & Clinical Neurosciences 1998, 52 Suppl:S344-347.
20. Rong Y, Glozier N, Luscombe GM, Davenport TA, Huang Y, Hickie IB: Improving knowledge and attitudes towards depression: a controlled trial among Chinese medical students. BMC Psychiatry 2011, 11:36.
21. Huang JJ, Chiovenda A, Shao Y, Ma HJ, Li HF, Good MJD: Low level of knowledge regarding diagnosis and treatment among inpatients with schizophrenia in Shanghai. Neuropsychiatric Disease and Treatment 2018, 14:185-191.
22. Yu Y, Liu ZW, Hu M, Liu HM, Yang JP, Zhou L, Xiao SY: Mental Health Help-Seeking Intentions and Preferences of Rural Chinese Adults. Plos One 2015, 10(11).
23. Chen S, Wu Q, Qi C, Deng H, Wang X, He H, Long J, Xiong Y, Liu T: Mental health literacy about schizophrenia and depression: a survey among Chinese caregivers of patients with mental disorder. BMC Psychiatry 2017, 17(1):89.
24. Wang J, He Y, Jiang Q, Cai J, Wang W, Zeng Q, Miao J, Qi X, Chen J, Bian Q et al: Mental health literacy among residents in Shanghai. Shanghai Arch Psychiatry 2013, 25(4):224-235.
25. Wu Q, Luo X, Chen S, Qi C, Long J, Xiong Y, Liao Y, Liu T: Mental health literacy survey of non-mental health professionals in six general hospitals in Hunan Province of China. PLoS ONE [Electronic Resource] 2017, 12(7):e0180327.
26. Liu W, Li YM, Peng Y: Mental health literacy: A cross-cultural study of American and Chinese bachelor of nursing students. Journal of Psychiatric and Mental Health Nursing 2018, 25(2):96-107.
27. Gong AT, Furnham A: Mental health literacy: Public knowledge and beliefs about mental disorders in mainland China. PsyCh Journal 2014, 3(2):144-158.
28. Li J, Li J, Huang YG, Thornicroft G: Mental health training program for community mental health staff in Guangzhou, China: effects on knowledge of mental illness and stigma. International Journal of Mental Health Systems 2014, 8.
29. Ni C, Ma L, Wang B, Yan Y, Huang Y, Wallen GR, Li L, Lang H, Hua Q: Neurotic disorders of general medical outpatients in Xi'an, China: knowledge, attitudes, and help-seeking preferences. Psychiatric Services 2014, 65(8):1047-1053.
30. Wei D, Tian Y, Gao H, Peng J, Tan Y, Li Y: Patient distress and emotional disclosure: a study of Chinese cancer patients. J Cancer Educ 2013, 28(2):346-351.
31. Rong Y, Luscombe GM, Davenport TA, Huang YQ, Glozier N, Hickie IB: Recognition and treatment of depression. Social Psychiatry and Psychiatric Epidemiology 2009, 44(8):636-642.
32. Yu Y, Hu M, Liu ZW, Liu HM, Yang JP, Zhou L, Xiao SY: Recognition of depression, anxiety, and alcohol abuse in a Chinese rural sample: a cross-sectional study. Bmc Psychiatry 2016, 16.
33. Sun L, Zhang J: Suicide Acceptability and Suicide Attempt: A Case-Control Study With Medically Serious Suicide Attempters Aged 15 to 54 Years in Rural China. Journal of Nervous and Mental Disease 2018, 206(9):694-698.
34. Yang BX, Stone TE, Davis SA: The effect of a community mental health training program for multidisciplinary staff. Archives of Psychiatric Nursing 2018, 32(3):413-417.
35. Tan L, Yang QH, Chen JL, Zou HX, Xia TS, Liu Y: The potential role of attitudes towards suicide between mental health status and suicidal ideation among Chinese children and adolescents. Child Care Health and Development 2017, 43(5):725-732.
36. Li J, Li J, Thornicroft G, Yang H, Chen W, Huang Y: Training community mental health staff in Guangzhou, China: evaluation of the effect of a new training model. In: BMC psychiatry. vol. 15; 2015: 263.
37. Ngai A, Bozza A, Zhang H, Chen C, Bennett P: Transition between cultures? Beliefs and attitudes of British and Chinese young adults living in China and the UK towards mental health disorders. International Journal of Culture & Mental Health 2014, 7(1):28-42.
38. 孟国荣,李学海,姚新伟, 等.1783名普通人群精神卫生知识知晓率调查结果及分析[J].上海精神医学,2005,17(z1):19-20. DOI:10.3969/j.issn.1002-0829.2005.z1.008.
39. 甘东,赵必华.194名高职院校学生自杀态度调查[J].中国校医,2010,24(2):132-134.
40. 蔡梅英,龚诚华,胡号应, 等.2011年广州市越秀区社区居民心理健康知识调查[J].预防医学论坛,2012,18(10):757-760.
41. 徐燕,李瑾,袁大伟, 等.2012年上海居民精神卫生知晓率及服务需求调查[J].江苏预防医学,2014,25(2):28-30. DOI:10.13668/j.issn.1006-9070.2014.02.010.
42. 董炳刚,冯春明,孙士华, 等.2013年山东省内3所大学学生自杀意念现状及其与心理扭力的关系[J].预防医学论坛,2015,21(6):426-429.
43. 顾美铮,王国宝.2047名老年人群及其照料者心理保健知识知晓率调查[J].上海精神医学,2005,17(z1):14-15. DOI:10.3969/j.issn.1002-0829.2005.z1.005.
44. 耿金根,王援越.2048名妇幼保健工作者心理保健知识知晓率的调查[J].上海精神医学,2005,17(z1):16. DOI:10.3969/j.issn.1002-0829.2005.z1.006.
45. 张国芳,赵军,饶顺曾, 等.2345名综合医院医务人员精神卫生知识知晓率的调查[J].上海精神医学,2005,17(z1):17-18. DOI:10.3969/j.issn.1002-0829.2005.z1.007.
46. 曾桂珍,胡媛,李东娜, 等.268名急诊医护人员对自杀患者的态度调查[J].护理学报,2017,24(20):38-40. DOI:10.16460/j.issn1008-9969.2017.20.038.
47. 高允锁,王小丹,孙鑫.3295名海南居民精神卫生与心理保健知识知晓情况分析[J].长江大学学报（自科版）,2017,14(20):61-64. DOI:10.3969/j.issn.1673-1409.2017.20.022.
48. 肖霄,吕春梅,凌冬兰, 等.465名产科医护人员对妊娠期妇女心理健康筛查知识态度及支持需求的水平分析[J].护理学报,2018,25(18):59-63. DOI:10.16460/j.issn1008-9969.2018.18.059.
49. 胡兰,陈晓颖.689名大学生自杀态度调查[J].上饶师范学院学报,2007,27(5):91-93. DOI:10.3969/j.issn.1004-2237.2007.05.020.
50. 常万琪,罗灏,戴莉.PDCA循环在预防肿瘤患者自杀管理中的应用[J].护理管理杂志,2015,15(3):201-203.
51. 马新,张悦,张媛, 等.安徽省妇女心理健康知识和精神疾病预防知识知晓情况[J].现代预防医学,2017,44(15):2792-2796.
52. 田梅,张勇,白珍, 等.保定市社区居民精神卫生知识认知态度和接触程度调查分析[J].首都医科大学学报,2012,33(3):377-380. DOI:10.3969/j.issn.1006-7795.2012.03.019.
53. 王海龙,霍耀芳,吕华, 等.保定市乡、村两级医院医务人员精神卫生知识知晓率及对精神疾病态度的调查[J].神经疾病与精神卫生,2014,14(5):486-489. DOI:10.3969/j.issn.1009-6574.2014.05.016.
54. 白珍,崔利军,张勇, 等.保定市与上海市居民心理卫生知识知晓率的调查研究[J].中国全科医学,2012,15(16):1878-1879. DOI:10.3969/j.issn.1007-9572.2012.06.031.
55. 马蕴宜,刘芳,黄森, 等.北京某社区居民抑郁症防治知识知晓情况调查[J].中国社会医学杂志,2012,29(4):269-271. DOI:10.3969/j.issn.1673-5625.2012.04.018.
56. 刘竞,闫芳,马辛, 等.北京市55周岁及以上人群精神障碍防治知识知晓率[J].中国健康心理学杂志,2013,21(11):1626-1628.
57. 宋珺,张婉奇,李文咏, 等.北京市朝阳区社区居民精神卫生知识与态度现况调查[J].中国健康心理学杂志,2013,21(11):1636-1638.
58. 闫芳,李文秀,靳伟国, 等.北京市高校大学生精神卫生知晓率及对精神疾病态度[J].中国健康心理学杂志,2013,21(6):822-825.
59. 何锐,李文秀,杨娜, 等.北京市海淀区居民的心理健康知识获得途径及对精神疾病的态度调查[J].职业与健康,2014,30(21):3063-3065,3068.
60. 何锐,李文秀,于玲, 等.北京市海淀区听讲座居民的精神卫生知识知晓差异分析[J].神经疾病与精神卫生,2014,14(3):293-295. DOI:10.3969/j.issn.1009-6574.2014.03.022.
61. 李红秋,崔宏亮,宋郡, 等.北京市某社区居民心理卫生知识调查及心理健康宣教干预模式研究[J].医学动物防制,2018,34(7):637-639. DOI:10.7629/yxdwfz201807006.
62. 程嘉,原岩波,文炳龙, 等.北京市社区医务人员精神卫生知识知晓度调查[J].中国心理卫生杂志,2016,30(1):18-22. DOI:10.3969/j.issn.1000-6729.2016.01.004.
63. 田亮,仪玉伟,靳玉宏, 等.北京市西城区居民心理健康知识知晓率调查研究[J].中国医学创新,2018,15(24):62-65. DOI:10.3969/j.issn.1674-4985.2018.24.017.
64. 陈雄,汪俊华.毕节市中学生自杀行为态度及影响因素分析[J].中国初级卫生保健,2018,32(2):53-56. DOI:10.3969/j.issn.1001-568X.2018.02.0021.
65. 高新义.不同民族城市公职人员精神疾病知晓率及态度[J].中国健康心理学杂志,2016,24(10):1441-1444. DOI:10.13342/j.cnki.cjhp.2016.10.001.
66. 高士元,费立鹏.不同人群对精神病的态度[J].中国心理卫生杂志,2001,15(2):107-109. DOI:10.3321/j.issn:1000-6729.2001.02.016.
67. 刘国义,高艳华,张艳娥,郝岩君.不同学历医学生的心理状况及对自杀态度:整群随机抽样调查[J].中国组织工程研究与临床康复,2007,(39):7855-7858.
68. 代光智,秦小荣.成都市基层医疗卫生机构精防人员精神卫生知识知晓率及培训需求调查[J].四川医学,2014,(6):750-752.
69. 黄敏,詹洪亮,何志文, 等.成都市新都区中学生心理卫生知识知晓率调查研究[J].中西医结合护理（中英文）,2018,4(6):86-88. DOI:10.11997/nitcwm.201806024.
70. 赵立琼,王周然,陈海燕, 等.成年男性抑郁、躯体化与自杀态度的相关性研究[J].华南国防医学杂志,2014,28(7):666-668. DOI:10.3969/j.issn.1009-2595.2014.07.015.
71. 张荣,张雷,杨青建, 等.承德市某校医学生精神卫生知识知晓率的调查与分析[J].中国健康心理学杂志,2016,24(4):497-500. DOI:10.13342/j.cnki.cjhp.2016.04.004.
72. 雷兰英,曹日芳.城市社区成年人对自杀的态度调查[J].浙江预防医学,2010,22(2):10-12. DOI:10.3969/j.issn.1007-0931.2010.02.004.
73. 阎红,刘书文,刘丽娟.城市社区人群自杀态度调查研究[J].中国卫生事业管理,2014,31(5):380-382.
74. 严保平,李建峰,栗克清, 等.城市与农村普通人群精神卫生知识知晓率及对精神疾病的态度[J].现代预防医学,2014,41(9):1636-1639.
75. 赵行暇,陈胜华,王颖初, 等.池州市育龄妇女心理健康和精神疾病预防知识知晓情况调查分析[J].安徽预防医学杂志,2017,23(5):325-328.
76. 王耀龙,徐英,王金华, 等.大学工科生、医学生与医务人员自杀态度的对照研究[J].苏州大学学报（医学版）,2006,26(5):767-769. DOI:10.3969/j.issn.1673-0399.2006.05.016.
77. 熊恋,郭雨薇,喻玲, 等.大学生的父母教养方式、自杀意念、自杀态度的关系[J].中国健康心理学杂志,2017,25(2):219-222. DOI:10.13342/j.cnki.cjhp.2017.02.017.
78. 吴蓉,何雪松.大学生的精神健康素养与专业求助倾向:以上海E校为例[J].华东理工大学学报(社会科学版),2013,28(05):101-108+116.
79. 蔡玲,林汉生,王声湧, 等.大学生对自杀行为的认知、态度及其影响因素的分析[J].中华疾病控制杂志,2010,14(10):980-983.
80. 唐永卿,杨宏飞.大学生对自杀态度的调查[J].中国心理卫生杂志,2003,17(4):282-283. DOI:10.3321/j.issn:1000-6729.2003.04.025.
81. 张冰,段彩彬.大学生对自杀态度及其心理健康调查分析[J].沈阳农业大学学报（社会科学版）,2014,16(6):697-700. DOI:10.3969/j.issn.1008-9713.2014.06.014.
82. 闫璐颖,谢玉诚.大学生精神卫生知识调查及探讨[J].考试周刊,2015,(75):154-154,155. DOI:10.3969/j.issn.1673-8918.2015.75.127.
83. 张建人,凌辉,谢健, 等.大学生人格障碍症状水平与自杀态度和自杀意念的关系[J].中国临床心理学杂志,2012,20(4):498-501.
84. 林玲,杨静,闫婉缔.大学生完美主义心理与自杀态度和自杀念头的相关研究[J].思想理论教育（上半月·综合版）,2006,(9):44-48. DOI:10.3969/j.issn.1007-192X.2006.09.011.
85. 江光荣,王铭.大学生心理求助行为研究[J].中国临床心理学杂志,2003,11(3):180-184. DOI:10.3969/j.issn.1005-3611.2003.03.007.
86. 任桂秀,叶云霞.大学生自杀的早期发现途径和方法[J].川北医学院学报,2007,22(1):72-74. DOI:10.3969/j.issn.1005-3697.2007.01.027.
87. 梁挺,张小远,徐雪花.大学生自杀“守门人”培训效果评价[J].中国学校卫生,2016,37(4):532-534. DOI:10.16835/j.cnki.1000-9817.2016.04.017.
88. 王玲,路仕容.大学生自杀态度、抑郁水平和自杀意念的研究[J].健康心理学杂志,2001,9(6):422-424. DOI:10.3969/j.issn.1005-1252.2001.06.011.
89. 罗京滨,杨玲,张文艺, 等.大学生自杀态度、自杀意念与生活事件及应付方式的调查研究[J].中国医学伦理学,2004,17(2):57-59. DOI:10.3969/j.issn.1001-8565.2004.02.027.
90. 王军,葛小锚,胡健, 等.大学生自杀态度、自杀意念与心理健康状况调查分析[J].中国行为医学科学,2005,14(9):830-831. DOI:10.3760/cma.j.issn.1674-6554.2005.09.036.
91. 白月薇.大学生自杀态度的研究[J].行政与法,2005,(6):66-67. DOI:10.3969/j.issn.1007-8207.2005.06.025.
92. 詹启生,王晗.大学生自杀态度对父母教养方式和自杀意念的中介作用[J].中国健康心理学杂志,2018,26(12):1876-1881. DOI:10.13342/j.cnki.cjhp.2018.12.033.
93. 杨玲,李丽丽.大学生自杀态度及其相关因素[J].中国社会医学杂志,2007,24(2):126-128,131. DOI:10.3969/j.issn.1673-5625.2007.02.020.
94. 田永果.大学生自杀态度调查研究[J].佳木斯教育学院学报,2014,(1):300-300,309.
95. 桑海云,咸大伟.大学生自杀态度相关研究[J].兰州教育学院学报,2014,(10):148-150. DOI:10.3969/j.issn.1008-5823.2014.10.065.
96. 关素珍,刘向阳,刘继文, 等.大学生自杀态度与家庭因素关系分析[J].中国学校卫生,2011,32(1):46-48.
97. 谢杏利,邹兵,黄中岩.大学生自杀态度与生活目的、生命意义感的关系[J].南方医科大学学报,2012,32(10):1482-1485. DOI:10.3969/j.issn.1673-4254.2012.10.022.
98. 刘薇,黄婉,舒勃桥.大学生自杀态度与生活事件的相关研究[J].成功(教育),2008,(05):205-206.
99. 傅晓荟,李丽萍.大学生自杀态度与抑郁情况研究[J].中国健康心理学杂志,2007,15(1):42-45. DOI:10.3969/j.issn.1005-1252.2007.01.012.
100. 李艳兰.大学生自我和谐应对方式与自杀态度的相关分析[J].中国学校卫生,2009,30(11):999-1001.
101. 郭文斌,张剑,冯小瑜, 等.大学新生自杀意念及其影响因素的现况调查[J].广西医科大学学报,2011,28(1):150-153. DOI:10.3969/j.issn.1005-930X.2011.01.057.
102. 张妍,孔繁昌,权珍桢, 等.地震灾区大学生生命价值观与自杀态度的调查分析[J].中华行为医学与脑科学杂志,2010,19(7):641-643. DOI:10.3760/cma.j.issn.1674-6554.2010.07.021.
103. 俞世伟,杨玲枝,和娟.独立学院大学生极端心理危机事件的调查[J].中国健康心理学杂志,2015,(4):606-610,611. DOI:10.13342/j.cnki.cjhp.2015.04.034.
104. 高向华,夏云.对法学与非法学专业大学生自杀态度的研究[J].现代预防医学,2007,34(7):1337-1338. DOI:10.3969/j.issn.1003-8507.2007.07.052.
105. 杨赛红.对精神分裂症患者家属进行健康教育的效果观察[J].当代护士（学术版）,2010,(6):49-51. DOI:10.3969/j.issn.1006-6411.2010.06.030.
106. 崔榕,郑刚,龚传鹏.对精神分裂症患者家属进行精神卫生知识教育的对照研究[J].中国民康医学（上半月）,2006,18(17):783-784. DOI:10.3969/j.issn.1672-0369.2006.17.044.
107. 吕国强,张霞,魏继祖, 等.对精神疾病和病人的认识、态度及对待方式的问卷调查[J].中国心理卫生杂志,2004,18(10):694,687. DOI:10.3321/j.issn:1000-6729.2004.10.007.
108. 王海龙,严保平,霍耀芳, 等.对口帮扶指导培训对保定市2013及2016年乡级医院医务人员精神卫生知识知晓率变化趋势的影响[J].中国健康心理学杂志,2018,26(2):178-181. DOI:10.13342/j.cnki.cjhp.2018.02.005.
109. 廖秀娥.对社区卫生服务中心医护人员精神卫生知识与技能培训的探讨[J].中国保健营养(上旬刊),2013,23(7):4094-4095. DOI:10.3969/j.issn.1004-7484(s).2013.07.780.
110. 张凯,崔萌,马军平, 等.对抑郁症患者自杀态度的研究[J].精神医学杂志,2008,21(6):409-410. DOI:10.3969/j.issn.1009-7201.2008.06.004.
111. 邱建国,宁洁,李小平.对重庆市部分大学生精神卫生知识相关问题的调查与分析[J].重庆医学,2009,38(15):1943-1945. DOI:10.3969/j.issn.1671-8348.2009.15.042.
112. 杨玲,杜鹃,杨仁登, 等.恩施市农村与城镇居民精神卫生知识知晓及服务需求状况调查[J].医学与社会,2017,30(6):63-65. DOI:10.13723/j.yxysh.2017.06.019.
113. 朱相华,梁光利,李娇, 等.儿童期虐待对中专学生自杀意念和自杀态度的影响[J].中国心理卫生杂志,2006,20(7):462-464. DOI:10.3321/j.issn:1000-6729.2006.07.014.
114. 梁红,费立鹏,张艳平, 等.发展中国家三城市非精神科门诊医务人员掌握抑郁症知识情况的初步调查[J].中国神经精神疾病杂志,2007,33(1):48-50. DOI:10.3969/j.issn.1002-0152.2007.01.012.
115. 邓云龙,唐秋萍,肖水源.非精神科医生对抑郁认识及相关因素的调查[J].中国行为医学科学,2001,10(1):63-64. DOI:10.3760/cma.j.issn.1674-6554.2001.01.036.
116. 方向,熊端华,陈旭先, 等.福建省居民精神卫生知识知晓率调查[J].海峡预防医学杂志,2010,16(5):30-31.
117. 王红英,栗克清,于雪竹, 等.妇幼保健机构医护人员对孕产妇产后精神分裂症的识别[J].中国健康心理学杂志,2014,22(2):164-166. DOI:10.13342/j.cnki.cjhp.2014.02.002.
118. 李铿,赵文莉,蒋霞, 等.甘肃省留守初中生自杀态度及影响因素分析[J].医学与社会,2017,30(7):62-65. DOI:10.13723/j.yxysh.2017.07.019.
119. 廖娟娟,郑亚楠,唐宏.赣州市大学生精神卫生知识知晓率调查分析[J].国外医学（医学地理分册）,2017,38(4):332-335. DOI:10.3969/j.issn.1001-8883.2017.04.006.
120. 夏云,高向华.高校师生对自杀态度的研究[J].现代预防医学,2006,33(2):225-226. DOI:10.3969/j.issn.1003-8507.2006.02.049.
121. 吴一玲,汪妍,丰辉, 等.高职医学生心理卫生知识知晓率调查分析[J].卫生职业教育,2013,31(16):108-110. DOI:10.3969/j.issn.1671-1246.2013.16.061.
122. 周红燕.高职院校大学生自杀态度与家庭功能相关研究[J].兰州石化职业技术学院学报,2015,(3):51-53. DOI:10.3969/j.issn.1671-4067.2015.03.016.
123. 张媛,胡小兵,程欣, 等.高中生自杀态度的特征及其与自杀意念[J].中国健康心理学杂志,2013,21(2):258-260.
124. 毛玉华.高中生自杀态度的现状分析——南昌市城区高中的调查[J].教育学术月刊,2009,(6):33-35.
125. 吴志敏.高中生自杀意念现状及其影响因素的调查分析[J].校园心理,2016,14(5):308-311. DOI:10.3969/j.issn.1673-1662.2016.05.007.
126. 姚丰菊,吕路线,秦志华, 等.个案管理培训对乡村精神卫生服务能力的影响[J].中国全科医学,2014,(22):2643-2647. DOI:10.3969/j.issn.1007-9572.2014.22.025.
127. 刘妍,陈宗阳.关于大学生自杀态度的调查研究[J].剑南文学,2013,(7):296-296. DOI:10.3969/j.issn.1006-026X.2013.07.200.
128. 林雪松,钟健敏,梁涯飞, 等.广东省大学生死亡、自杀态度及死亡教育需求调查[J].医学与社会,2012,25(10):79-81. DOI:10.3870/YXYSH.2012.10.025.
129. 陆建兰.广西大学生自杀态度与自我意识自我和谐的关系[J].中国学校卫生,2018,39(6):900-902. DOI:10.16835/j.cnki.1000-9817.2018.06.029.
130. 黎泽明,黎燕宁,冯启明,江南,罗红叶.广西农村基层精防人员精神卫生与心理保健知识及精神疾病态度现状调查[J].中国全科医学,2016,19(S1):315-318.
131. 胡号应,颜瑜章,陈力鸣, 等.广州市城乡居民的心理健康知识知晓率及对精神疾病的态度[J].中国心理卫生杂志,2012,26(1):30-35. DOI:10.3969/j.issn.1000-6729.2012.01.007.
132. 杨春,李晓珍,陈庆良, 等.广州市黄埔区居民精神卫生知识知晓率现状调查[J].中国健康教育,2013,29(4):377-379.
133. 潘胜茂,周英,唐省三, 等.广州市居民对精神疾病态度及其影响因素研究[J].医学与哲学,2016,37(3):20-22,30. DOI:10.12014/j.issn.1002-0772.2016.02a.05.
134. 黄昱,张碧艳,林耀琪, 等.广州市理工院校大学生自杀态度的调查分析[J].中国健康教育,2007,23(9):650-652. DOI:10.3969/j.issn.1002-9982.2007.09.004.
135. 邝仕源,黄懿炘,宋世坤, 等.广州市某高校临床医学研究生精神卫生知识水平及对精神疾病的态度[J].职业与健康,2017,33(20):2848-2851.
136. 吴婉云,李娜玲,陈秀革.广州市社区卫生服务机构医务人员精神卫生知识调查[J].现代预防医学,2015,42(22):4126-4129.
137. 罗之勇,刘雪珍,钟乃良.桂西北大学生生命意义感与自杀态度的调查研究[J].学术论坛,2012,35(5):187-191. DOI:10.3969/j.issn.1004-4434.2012.05.042.
138. 佟海龙,焦辛妮,顾星博, 等.哈尔滨市社区居民精神卫生知识认知及对患者态度的调查及影响因素分析[J].实用预防医学,2015,22(10):1199-1203. DOI:10.3969/j.issn.1006-3110.2015.010.014.
139. 王海莲,武丽杰,孙彩虹, 等.哈尔滨市中学生心理健康状况和自杀态度的相关性研究[J].中国学校卫生,2006,27(6):487-489. DOI:10.3969/j.issn.1000-9817.2006.06.027.
140. 罗建强,陈杨.海洛因依赖者自杀态度调查[J].中国药物滥用防治杂志,2011,17(5):286,309. DOI:10.3969/j.issn.1006-902X.2011.05.014.
141. 陈爱华,汪红梅,徐项坤, 等.杭州市初级保健人员自杀态度与自杀知识调查[J].中国农村卫生事业管理,2010,30(9):782-784.
142. 孙晓花,宋海东,金翠梅, 等.杭州市人群精神卫生核心信息知晓率调查[J].浙江预防医学,2010,22(7):88-89. DOI:10.3969/j.issn.1007-0931.2010.07.043.
143. 陈树林,王义强.杭州市中学生自杀态度分析[J].中国学校卫生,2009,30(8):710-712.
144. 张盼,赵雅宁,刘海娟, 等.河北省农村居民心理健康知识知晓率及对精神疾病态度调查[J].中国卫生事业管理,2013,30(10):777-780. DOI:10.3969/j.issn.1004-4663.2013.10.019.
145. 张士巧,乔云栓,乔翠, 等.衡水市城乡居民精神卫生知识认知现状及行为趋向分析[J].中国健康心理学杂志,2016,24(2):182-186. DOI:10.13342/j.cnki.cjhp.2016.02.006.
146. 崔卫东,乔云栓,张士巧, 等.衡水市乡镇卫生院医务人员精神卫生知识现状[J].中国健康心理学杂志,2015,(4):495-497,498. DOI:10.13342/j.cnki.cjhp.2015.04.005.
147. 何倩,郭蕊,时俊新, 等.湖北省两市初一学生毒品滥用认知和行为调查[J].中华流行病学杂志,2008,29(4):329-332. DOI:10.3321/j.issn:0254-6450.2008.04.005.
148. 严鼎,陈钰,高远, 等.湖北省某高校大学生自杀态度及领悟社会支持状态[J].健康必读（中旬刊）,2012,11(10):60-61.
149. 冯珊珊,肖水源,白丽琼.湖南部分医学院学生自杀认知水平的调查研究[J].中国临床心理学杂志,2003,11(3):219-220. DOI:10.3969/j.issn.1005-3611.2003.03.022.
150. 付文彬,罗邦安,刘学军, 等.湖南省乡镇卫生院精神疾病防治人员精神卫生知识知晓情况调查[J].神经疾病与精神卫生,2015,(4):364-367. DOI:10.3969/j.issn.1009-6574.2015.04.012.
151. 贾金铃,周位珍,何清清.互动达标理论在双相情感障碍患者中的应用效果[J].护理实践与研究,2018,15(14):146-147. DOI:10.3969/j.issn.1672-9676.2018.14.060.
152. 耿笑微.护理本科学生对精神障碍患者态度的调查分析[J].中华护理杂志,2015,50(10):1217-1221. DOI:10.3761/j.issn.0254-1769.2015.10.013.
153. 姜蕾,刘红蕾,彭中华, 等.基层妇女保健人员产后抑郁相关知识培训的效果评价[J].中国妇幼保健,2018,33(4):880-883. DOI:10.7620/zgfybj.j.issn.1001-4411.2018.04.53.
154. 辛阔林,张康莉,张晓明.基层官兵自杀认识和态度与心理健康状况的相关性分析[J].实用医药杂志,2009,26(11):67-69. DOI:10.3969/j.issn.1671-4008.2009.11.045.
155. 林汉,刘小兵,孙志国, 等.集训期新兵自杀态度的心理社会相关因素[J].中华行为医学与脑科学杂志,2016,25(1):76-81. DOI:10.3760/cma.j.issn.1674-6554.2016.01.016.
156. 张璟,王文军,宋烨, 等.济宁市居民精神卫生知识知晓率调查[J].济宁医学院学报,2009,32(4):282-284. DOI:10.3969/j.issn.1000-9760.2009.04.021.
157. 赵萍,陶婵梅,李雪芳, 等.健康教育社区干预对慢性精神分裂症患者及家属的作用[J].中国健康心理学杂志,2018,26(6):804-807. DOI:10.13342/j.cnki.cjhp.2018.06.002.
158. 林曙光,林熙,钟文玲, 等.将乐县居民精神卫生知识知晓率调查[J].海峡预防医学杂志,2008,14(6):24-26. DOI:10.3969/j.issn.1007-2705.2008.06.010.
159. 袁燕子,王红兰.阶段式的依从性干预措施在精神分裂症患者中的应用[J].中国临床护理,2018,10(5):415-417. DOI:10.3969/j.issn.1674-3768.2018.05.015.
160. 余存霞,叶晓玲,鲍丽丽, 等.金安区妇女心理健康知识和精神卫生知识知晓情况调查报告[J].安徽预防医学杂志,2018,24(2):142-143.
161. 齐玉梅,王生锋,王桂华.荆门市城市居民精神卫生知识掌握及获取途径的调查分析[J].中国实用护理杂志,2010,26(26):9-11. DOI:10.3760/cma.j.issn.1672-7088.2010.09.045.
162. 高雪屏,李凌江,陈晋东.精神病患者家属的精神卫生知识需求和态度调查[J].中国行为医学科学,2004,13(5):550-551. DOI:10.3760/cma.j.issn.1674-6554.2004.05.040.
163. 李立华,李穗云,黄丽红, 等.精神病患者家属精神卫生知识水平及结构调查[J].护理学报,2006,13(6):19-21. DOI:10.3969/j.issn.1008-9969.2006.06.008.
164. 曾承.精神病患者求医行为的影响因素分析[J].中国医药导报,2011,08(3):140-141. DOI:10.3969/j.issn.1673-7210.2011.03.078.
165. 王文伶,卢健宁,伍雪晶.精神分裂症患者家属的精神卫生知识了解和需求的调查#[J].中国医药导刊,2012,(z1):370. DOI:10.3969/j.issn.1009-0959.2012.z1.292.
166. 焦桂花,谢博,朱闵敏.精神分裂症患者家属对精神障碍知晓情况的调查与分析[J].教育教学论坛,2017,(27):74-75.
167. 张启文,黄胜,蒋陆平, 等.精神分裂症患者家属精神卫生知识及感知歧视调查[J].中国医药导报,2014,(19):103-106.
168. 焦亚辉,王丽娜,周郁秋, 等.精神分裂症恢复期患者与照顾者精神疾病相关知识及行为调查[J].广东医学,2017,38(17):2680-2683. DOI:10.3969/j.issn.1001-9448.2017.17.027.
169. 潘胜茂,周英,林建睽, 等.精神科护士对精神疾病态度及影响因素分析[J].护理学报,2013,(15):72-74. DOI:10.3969/j.issn.1008-9969.2013.15.028.
170. 赵蓉,王金爱,赵晓华, 等.精神科护士精神疾病知识与态度调查[J].当代护士（下旬刊）,2015,(7):21-23,24.
171. 朱文标,陈策,郑丽丹, 等.精神科医务人员对精神卫生知识认知调查[J].医院管理论坛,2018,35(1):46-48. DOI:10.3969/j.issn.1671-9069.2018.01.014.
172. 陈淑宝,吴秋霞,王绪轶, 等.精神科照料者的抑郁症及广泛性焦虑症知识知晓状况调查[J].中国心理卫生杂志,2018,32(11):923-925. DOI:10.3969/j.issn.1000-6729.2018.11.007.
173. 梁敉宁,李乐之,陈琼妮, 等.精神卫生知识培训对综合性医院非精神科护士干预效果研究[J].当代护士（专科版）,2014,(9):29-30,31.
174. 潘惠君,郭红,陈宏美, 等.精神卫生知识普及情况及其相关因素调查研究[J].山西医药杂志,2012,41(23):1195-1197.
175. 孟宪锋.精神卫生知识宣教对精神疾病患病率的影响分析[J].医学信息,2014,(6):350-350. DOI:10.3969/j.issn.1006-1959.2014.06.427.
176. 张宏伟.精神卫生知识知晓情况及对待精神病人态度、方式调查[J].中国健康心理学杂志,2011,19(11):1307-1309.
177. 赵成,李官鸿,于碧涛, 等.九龙坡区居民精神卫生知识知晓率及服务需求状况分析[J].中国健康心理学杂志,2010,18(2):134-136.
178. 史瑞洁,刘华,李琪, 等.军队干休所保健人员精神卫生知识及态度调查[J].中国健康心理学杂志,2018,26(5):661-664. DOI:10.13342/j.cnki.cjhp.2018.05.007.
179. 马娇,李琪,宋咪, 等.军医大学本科生精神卫生知晓情况及其影响因素探析[J].护理实践与研究,2018,15(6):132-135. DOI:10.3969/j.issn.1672-9676.2018.06.054.
180. 韩慧琴,曾勇,赵旭东, 等.昆明市普通人群精神卫生知识知晓率调查[J].中国健康心理学杂志,2008,16(11):1274-1277.
181. 张媛,刘倩倩.昆明医科大学医学生自杀企图和自杀态度状况调查分析[J].昆明医科大学学报,2013,(12):30-32,36.
182. 张少华,刘忠华,于文平.莱芜市莱城农村社区人群心理健康认知调查[J].中国健康教育,2002,18(10):653-654. DOI:10.3969/j.issn.1002-9982.2002.10.019.
183. 杨小龙,吕红波,焦歆益, 等.兰州市城乡居民对精神疾病态度的现状调查[J].临床精神医学杂志,2017,27(6):388-390. DOI:10.3969/j.issn.1005-3220.2017.06.009.
184. 杨小龙,霍小宁,焦歆益, 等.兰州市城乡居民精神卫生知识知晓率调查与分析[J].国外医学（医学地理分册）,2016,37(1):57-59. DOI:10.3969/j.issn.1001-8883.2016.01.013.
185. 杨小龙,霍小宁,吕红波, 等.兰州市大中学生精神卫生知识知晓率调查[J].中国民康医学,2014,(5):76-78. DOI:10.3969/j.issn.1672-0369.2014.05.040.
186. 姜晓梅,张兰.兰州市中学生心理健康状况及自杀态度的研究[J].湖南医科大学学报（社会科学版）,2009,11(4):228-231.
187. 李光华,陈文明,鲁文兴, 等.澜沧县拉祜族、爱尼族对自杀态度的调查[J].临床精神医学杂志,2005,15(4):238-239. DOI:10.3969/j.issn.1005-3220.2005.04.038.
188. 李建峰,严保平,栗克清, 等.老年期常见精神疾病的症状和预防知识调查[J].中国健康心理学杂志,2014,22(3):343-345. DOI:10.13342/j.cnki.cjhp.2014.03.011.
189. 谢红平,唐铭民,徐双, 等.老年期常见精神疾病症状和预防知识知晓率调查[J].养生保健指南,2018,(14):23-24.
190. 杨宁,颜瑜章,陈力鸣, 等.老年人及其照料者对老年期常见精神障碍症状和预防知识的知晓率[J].中国心理卫生杂志,2012,26(5):327-331. DOI:10.3969/j.issn.1000-6729.2012.05.002.
191. 陈万海,彭涛,杨艳杰, 等.理工科与医科大学生对自杀态度的研究[J].健康心理学杂志,2001,9(4):273-274. DOI:10.3969/j.issn.1005-1252.2001.04.024.
192. 谭文艳,于瑞丽,杨晓东, 等.林芝地区精神卫生工作者精神卫生知识知晓率及其对精神疾病的态度[J].四川精神卫生,2017,30(6):546-550. DOI:10.11886/j.issn.1007-3256.2017.06.015.
193. 于品.临床护理路径应用在狂躁抑郁症患者护理中的效果观察[J].中国现代药物应用,2018,12(3):182-183. DOI:10.14164/j.cnki.cn11-5581/r.2018.03.112.
194. 徐艳.临床护理路径用于精神分裂症患者健康教育的实施效果评价[J].医学食疗与健康,2018,(5):149.
195. 唐勇,周亮,肖水源, 等.浏阳市乡村医务人员自杀、中毒急救与精神病学知识调查[J].临床精神医学杂志,2005,15(4):235-237. DOI:10.3969/j.issn.1005-3220.2005.04.036.
196. 周志鹏,阮雪玲,蓝永贵, 等.龙岩市大学生精神卫生现况调查[J].海峡预防医学杂志,2009,15(3):32-33.
197. 郭爱鸽,易利民.聋人大学生自杀态度调查及对策研究[J].科教导刊,2016,(22):188-190. DOI:10.16400/j.cnki.kjdks.2016.08.089.
198. 徐爱枝,严双琴,钱芳, 等.马鞍山市621名孕妇焦虑抑郁、精神卫生知识知晓情况及对精神疾病态度的现况调查[J].中国妇幼保健,2017,32(10):2198-2200. DOI:10.7620/zgfybj.j.issn.1001-4411.2017.10.54.
199. 邹敏.锚定信息对青少年自杀态度的影响[J].中国学校卫生,2015,36(10):1554-1556.
200. 朱自立,许燕,单雯, 等.媒体自杀报道对象对大学生自杀态度的影响[J].中国健康心理学杂志,2008,16(3):299-301. DOI:10.3969/j.issn.1005-1252.2008.03.025.
201. 李清金,黄宣,卢武生, 等.绵阳市农村社区人群精神卫生知识知晓率现状调查[J].中国医药导报,2014,(36):91-95.
202. 刘知源,谢红梅,丁永涛, 等.某部军人心理卫生知识知晓率调查[J].解放军医药杂志,2011,23(1):62-64. DOI:10.3969/j.issn.2095-140X.2011.01.036.
203. 荆春霞,王声湧,杨光, 等.某高校大学生自杀态度及影响因素分析[J].中国公共卫生,2008,24(8):913-915.
204. 温多红,夏云.某高校医专学生对待自杀的态度[J].中国学校卫生,2005,26(7):578-579. DOI:10.3969/j.issn.1000-9817.2005.07.023.
205. 杨万龄,傅炜昶,罗盈怡, 等.某高职院校护生自杀态度调查[J].中国学校卫生,2013,34(8):1002-1003.
206. 夏云.某高职院校医学生对自杀的态度[J].中国学校卫生,2006,27(2):158-159. DOI:10.3969/j.issn.1000-9817.2006.02.033.
207. 孔刚,孙业桓,杨林胜, 等.某医学院校大学生自杀态度的社会心理相关因素探讨[J].蚌埠医学院学报,2012,37(10):1240-1244. DOI:10.3969/j.issn.1000-2200.2012.10.039.
208. 姜杨,吕月娣,徐瑾, 等.某综合性医院低年资护士精神卫生知识知晓状况调查[J].浙江医学教育,2011,10(2):36-38. DOI:10.3969/j.issn.1672-0024.2011.02.015.
209. 郑亚楠,伍龙,魏泽红, 等.某综合医院护士精神卫生知识知晓率调查[J].中国现代医学杂志,2016,26(9):106-109. DOI:10.3969/j.issn.1005-8982.2016.09.023.
210. 喻芳,郭明,卢小勇, 等.南昌市326名医学硕士研究生自杀态度及其影响因素研究[J].现代预防医学,2012,39(2):281-283.
211. 雍那,任玉玲,王春元, 等.南充市中学生心理求助状况及专业心理求助态度[J].中国学校卫生,2018,39(8):1253-1255. DOI:10.16835/j.cnki.1000-9817.2018.08.041.
212. 林代琼,李长英,曾连, 等.内江市公务员抑郁症相关知识知晓现状的调查研究[J].四川医学,2017,38(9):1021-1024. DOI:10.16252/j.cnki.issn1004-0501-2017.09.012.
213. 王变云 朱晓卓.宁波市大学生精神卫生知识知晓率及对精神疾病的态度[J].辽宁医学院学报：社会科学版,2014,12(3).
214. 李建芳.农村老年人对自杀的态度调查[J].上海精神医学,2010,22(2):103-104. DOI:10.3969/j.issn.1002-0829.2010.02.013.
215. 毛文君,向孟泽,冉茂盛, 等.农村社区精神卫生服务中初级卫生人员培训的对照研究[J].临床精神医学杂志,1998:24-25.
216. 王东芳,贾存显,路长飞.农村社区人群自杀相关态度的性别比较[J].中国卫生事业管理,2011,28(7):539-541,553. DOI:10.3969/j.issn.1004-4663.2011.07.023.
217. 韩红霞,薛将,孙贤斌, 等.农村社区医生对老年抑郁症的知识、态度与管理自信心[J].中国农村卫生事业管理,2016,36(8):1039-1042.
218. 王琳琳,贾存显,邱惠敏, 等.农村自杀未遂人群对自杀行为的认知与态度研究[J].山东大学学报（医学版）,2012,50(9):118-123. DOI:10.6040/j.issn.1671-7554.2012.09.026.
219. 韩国玲,杜欣柏,刘文华, 等.青南藏区精神疾病患者求医行为及影响因素[J].临床精神医学杂志,2009,19(5):314-314.
220. 陶婵梅,丁淑艳,李雪芳,诸雅芳.青浦区社区居民精神卫生知晓率调查分析[J].健康教育与健康促进,2014,9(04):296-298.
221. 吴艳,许冬梅,张海娟, 等.全国部分地区精神专科医院和综合医院护士抑郁症知识和态度的调查[J].中华现代护理杂志,2017,23(15):1953-1959. DOI:10.3760/cma.j.issn.1674-2907.2017.15.001.
222. 张振兰,姚绍敏,王岚, 等.认知行为干预对老年抑郁障碍自杀态度影响的研究[J].护士进修杂志,2012,27(18):1641-1643. DOI:10.3969/j.issn.1002-6975.2012.18.004.
223. 徐敬文,金芳,温博贤.认知行为干预对抑郁症患者睡眠质量和自杀态度的影响[J].中国民康医学,2013,25(13):35-36. DOI:10.3969/j.issn.1672-0369.2013.13.009.
224. 施忠英,曹新妹,诸海英, 等.认知行为干预对抑郁症患者抑郁情绪和自杀态度影响的研究[J].上海护理,2007,7(6):25-27. DOI:10.3969/j.issn.1009-8399.2007.06.006.
225. 陈莹.厦门市居民精神卫生知识知晓率调查[J].中国健康心理学杂志,2011,19(10):1202-1203.
226. 贾存显,庄茂强,王健.山东省西部农村居民主观幸福感与自杀态度的关系[J].山东大学学报（医学版）,2012,50(8):128-132. DOI:10.6040/j.issn.1671-7554.2012.08.029.
227. 邓怀丽,王自强.山西省太原市精神卫生防治管理人员精神卫生知识知晓率调查[J].中国基层医药,2014,(24):3827-3828,3829. DOI:10.3760/cma.j.issn.1008-6706.2014.24.066.
228. 朱文,崔莹莹,韩凌, 等.汕头市重性精神疾病患者家属精神卫生知识知晓现状[J].汕头大学医学院学报,2018,31(2):121-124.
229. 刘彩萍,谢斌,韩慧琴, 等.上海、昆明两城区居民精神卫生知识知晓与服务需求对比研究[J].上海精神医学,2008,20(3):152-155. DOI:10.3969/j.issn.1002-0829.2008.03.008.
230. 王琰,何燕玲,王静夷, 等.上海和北京及长沙三个城市焦虑障碍和抑郁症的大众知晓度调查[J].中华精神科杂志,2015,48(4):220-226. DOI:10.3760/cma.j.issn.1006-7884.2015.04.007.
231. 卢艳,曾庆枝,赵晓光, 等.上海嘉定区老年人精神卫生知识知晓状况调查[J].国际精神病学杂志,2018,45(3):487-490.
232. 仇剑崟,谢斌.上海社区居民精神卫生知识知晓和服务需求调查[J].中国健康心理学杂志,2005,13(2):81-85. DOI:10.3969/j.issn.1005-1252.2005.02.001.
233. 蒋怡华,卜时明,沈剑, 等.上海市闵行区普通市民精神卫生知识知晓率调查[J].上海预防医学,2007,19(6):281-282. DOI:10.3969/j.issn.1004-9231.2007.06.024.
234. 袁勤,陆红英,王爱华, 等.上海市浦东新区南汇地区抑郁障碍患者自杀态度及影响因素[J].职业与健康,2017,33(23):3264-3267,3271.
235. 张静雅,王伟,曾庆枝, 等.上海市社区医护人员精神卫生知识的现状及影响因素[J].中国初级卫生保健,2018,32(1):28-30. DOI:10.3969/j.issn.1001-568X.2018.01.0010.
236. 吴海苏,徐勇,徐一峰, 等.上海市社区医生对老年抑郁症态度和知识的问卷调查[J].上海精神医学,2007,19(6):341-344. DOI:10.3969/j.issn.1002-0829.2007.06.006.
237. 孟国荣,姚新伟,朱紫青,张明园.上海市市民精神卫生知识知晓率调查:2697份问卷分析[J].上海精神医学,2002,(S1):56-57.
238. 徐妹,张国芳,周卿, 等.上海市徐汇区某社区老年人群及其照料者精神卫生知识知晓率调查[J].中国初级卫生保健,2013,27(4):17-19. DOI:10.3969/j.issn.1001.568X.2013.04.0006.
239. 张世伟,凤玉英.上海市颛桥镇居民精神卫生知识知晓率调查[J].中国校医,2010,(4):293-294.
240. 刘玉娟,韩立娟,王英杰.社会支持与大学生自杀态度的相关研究[J].唐山师范学院学报,2011,33(1):139-142. DOI:10.3969/j.issn.1009-9115.2011.01.043.
241. 司敏,谭继平,杨珊, 等.社区护士自杀相关态度及教育干预研究[J].护理学杂志,2015,30(10):98-100,105. DOI:10.3870/hlxzz.2015.10.098.
242. 司敏,谭继平,杨珊, 等.社区护士自杀相关态度及教育干预研究[J].护理学杂志,2015,30(10):98-100,105. DOI:10.3870/hlxzz.2015.10.098.
243. 周小青,姚丰菊.社区精神分裂症照顾者精神卫生知识知晓率及生活质量状况调查[J].临床心身疾病杂志,2014,(4):97-98,120. DOI:10.3969/j.issn.1672-187X.2014.04.035-0097-03.
244. 王洋,赵大磊.社区居民心理健康知识知晓率调查分析[J].中国民康医学,2016,28(6):104-105. DOI:10.3969/j.issn.1672-0369.2016.06.051.
245. 陈树林,许百华,程荑, 等.社区全科医生对抑郁症态度调查[J].中国公共卫生,2010,26(1):114-115.
246. 赵晶,冯玫.社区全科医生精神卫生及相关常见精神疾病的知晓率调查[J].中西医结合心脑血管病杂志,2016,14(9):1040-1044. DOI:10.3969/j.issn.1672-1349.2016.09.041.
247. 王政科,孙翠琴,傅荷芬, 等.社区卫生服务中心医务人员精神卫生知识的调查[J].上海精神医学,2003,15(1):10-12. DOI:10.3969/j.issn.1002-0829.2003.01.004.
248. 包小君,陈爱华.社区卫生服务中心医务人员精神卫生知识培训效果评价[J].中国基层医药,2011,18(9):1200-1201. DOI:10.3760/cma.j.issn.1008-6706.2011.09.023.
249. 姜莹,陶旻枫,王辰.社区医生产后抑郁知识及相关工作的现况调查[J].中国妇幼健康研究,2015,26(6):1291-1293. DOI:10.3969/j.issn.1673-5293.2015.06.062.
250. 邓秀良,唐建军,张泉水, 等.深圳市宝安区劳务工抑郁症相关精神卫生知识调查[J].中国社会医学杂志,2014,(2):100-102. DOI:10.3969/j.issn.1673-5625.2014.02.010.
251. 刘小瑜,吴斌,王倩.深圳市中学生预防精神活性物质滥用干预效果评价[J].中国公共卫生,2016,32(3):291-294. DOI:10.11847/zgggws2016-32-03-10.
252. 刘静,谢杏利.生命意义感和幸福感对研究生自杀态度的影响[J].现代预防医学,2015,42(7):1251-1253,1275.
253. 王文伶,张启文,黄振洲.师范院校体育专业大学生身心健康状况及自杀态度研究[J].河北医药,2013,(17):2669-2670. DOI:10.3969/j.issn.1002-7386.2013.17.063.
254. 孙岩.石家庄市城市居民精神卫生知识知晓率及对精神疾病态度的调查[D].河北:河北医科大学,2015.
255. 胡德英,熊宇,杨雪娇, 等.实习护生对综合医院住院病人自杀预防的认知调查[J].护理研究,2017,31(2):167-171. DOI:10.3969/j.issn.1009-6493.2017.02.011.
256. 阿怀红,廖东升,郭应丽, 等.首次住专科医院精神障碍患者家属求医态度、心理状况及家庭关系调查[J].临床精神医学杂志,2015,(5):340-341.
257. 王瑛,翟丽,李凯, 等.首发精神分裂症患者家属心理健康与分裂症知晓情况的关系[J].中国医药导报,2014,(36):88-90,95.
258. 周爽,余新年,何华.硕士研究生自杀态度的调查[J].四川精神卫生,2007,20(2):92-94. DOI:10.3969/j.issn.1007-3256.2007.02.010.
259. 周海云,周秀娟,金丽霓, 等.思维导图在老年抑郁症患者健康教育中的应用[J].护理与康复,2018,17(8):86-89. DOI:10.3969/j.issn.1671-9875.2018.08.029.
260. 陆艳红,刘晓瑛,宋媛.苏州市社区居民对儿童精神卫生知识知晓率调查[J].中国妇幼保健,2010,25(14):1969-1971.
261. 李卫东,孙永合,司文霞, 等.泰安地区城乡初中生心理卫生知识知晓率调查分析[J].精神医学杂志,2015,(4):254-257. DOI:10.3969/j.issn.2095-9346.2015.04.005.
262. 李秀红,张雪芹,张慧, 等.泰安市社区居民精神卫生知识知晓率调查研究[J].中华行为医学与脑科学杂志,2015,24(1):78-79. DOI:10.3760/cma.j.issn.1674-6554.2015..
263. 刘玉娟,张健,姚晓青.唐山市大学生自杀态度与生活事件相关研究[J].中国健康心理学杂志,2009,17(9):1079-1081.
264. 柳宝祥,刑淑芳,李国华, 等.体育专业大学生对自杀态度的调查研究[J].现代预防医学,2008,35(6):1115-1117. DOI:10.3969/j.issn.1003-8507.2008.06.055.
265. 王砚华,崔炳喜,杜长军, 等.天津市城市居民对抑郁症的认识和态度调查[J].临床护理杂志,2010,09(3):2-4. DOI:10.3969/j.issn.1671-8933.2010.03.001.
266. 黄彦,徐广明,尹慧芳, 等.天津市社区居民精神卫生知识知晓率及相关因素[J].国际精神病学杂志,2015,42(4):23-26.
267. 王小全,李刚,丁志杰, 等.天水市城市与农村普通人群精神卫生知识知晓率及对精神疾病的态度[J].四川精神卫生,2017,30(2):168-174. DOI:10.11886/j.issn.1007-3256.2017.02.017.
268. 陈雄,汪俊华,汪小斌, 等.网络游戏对贵州省中学生自杀态度的影响[J].中国学校卫生,2018,39(2):226-228. DOI:10.16835/j.cnki.1000-9817.2018.02.020.
269. 胥爱萍,葛茂宏,张功法, 等.潍坊市城乡居民精神卫生知识知晓率问卷调查分析[J].精神医学杂志,2010,23(6):457-458. DOI:10.3969/j.issn.1009-7201.2010.06.023.
270. 高伟博,葛茂宏,张功法, 等.潍坊市城乡居民心理卫生知识知晓率调查[J].中国民康医学,2016,(2):89-92. DOI:10.3969/j.issn.1672-0369.2016.02.047.
271. 贾黎斋,王宇中,赵山明.卫校女生自杀态度调查[J].中国心理卫生杂志,2006,20(2):107-108. DOI:10.3321/j.issn:1000-6729.2006.02.011.
272. 郭宁,王肖川.未婚首发精神分裂症患者家属的就医态度及心理状态调查[J].中国民康医学,2017,29(3):63-65. DOI:10.3969/j.issn.1672-0369.2017.03.030.
273. 李虹,黄悦勤.我国科研院所研究生自杀意念影响因素研究[J].中国健康心理学杂志,2009,17(8):963-966.
274. 芮婷婷,吴炜林,胡翠荣, 等.芜湖市育龄妇女心理健康知识知晓率调查及影响因素分析[J].现代预防医学,2016,43(22):4096-4098,4109.
275. 肖强,李小红,王珍, 等.武汉某校大学生精神卫生知识抽样调查[J].中国健康心理学杂志,2014,22(3):348-350. DOI:10.13342/j.cnki.cjhp.2014.03.013.
276. 杨玲花,梅红彬,张帆, 等.武汉市江汉区卫生服务机构医务人员精神卫生知识知晓状况及其影响因素[J].职业与健康,2017,33(10):1342-1344,1352.
277. 陈静,毛艳丽,张婕, 等.武汉市民办院校大学生精神卫生知晓率及对精神疾病态度的调查[J].青年时代,2018,(13):172-173.
278. 周洋,陈文材,李文学, 等.武汉市社区医务人员精神卫生知识知晓率调查[J].现代预防医学,2017,44(8):1425-1427,1436.
279. 张建芳,陈红辉,钟宝亮, 等.武汉市中学生精神卫生知识知晓情况调查[J].中国健康教育,2010,26(8):609-611.
280. 梁小平,吴斌,朱晓静, 等.西安市老年人群及其照料者心理卫生知识知晓率调查[J].中国健康心理学杂志,2012,20(1):19-21.
281. 梁小平,朱晓静,刘婷, 等.西安市社区居民精神卫生知识知晓率现况调查[J].中国健康心理学杂志,2011,19(10):1200-1202.
282. 陈强,韦波,唐海宁, 等.乡村医生精神疾病防治康复知识培训效果分析[J].应用预防医学,2010,16(5):289-290. DOI:10.3969/j.issn.1673-758X.2010.05.016.
283. 谷灵犀,齐振标,贾存显.乡村医生自杀认知、态度与自尊的关系[J].中国心理卫生杂志,2018,32(2):118-124. DOI:10.3969/j.issn.1000-6729.2018.02.006.
284. 徐荣静,于伟文,汤明燕, 等.心境障碍患者家属的疾病知识掌握情况的调查分析[J].中外医疗,2017,36(8):170-172,195. DOI:10.16662/j.cnki.1674-0742.2017.08.170.
285. 刘旺发,黄俊玲,周隽, 等.心理干预对初中生心理卫生知识知晓率的影响[J].中国校医,2017,31(6):413,416.
286. 张曼华,石扩,张婉奇, 等.心理健康宣教对社区居民精神卫生知晓率的影响[J].中国健康心理学杂志,2015,23(11):1613-1616. DOI:10.13342/j.cnki.cjhp.2015.11.005.
287. 常向东,陈勇,金霞芳, 等.心理卫生进社区对居民心理干预前后的比较研究[J].中国民康医学,2012,24(1):1-3,7. DOI:10.3969/j.issn.1672-0369.2012.01.001.
288. 陈志强,魏绪伟,毕存箭.新疆师范类高校维吾尔族学生自杀态度及影响因素[J].中国学校卫生,2014,35(8):1220-1222.
289. 张伟波,张国芳,沈文龙, 等.徐汇区精神疾患家属对精神病知、信、行的调查分析[J].中国初级卫生保健,2010,24(5):22-24. DOI:10.3969/j.issn.1001-568X.2010.05.011.
290. 王大江,周刚,温红伟, 等.许昌市城乡居民自杀态度及影响因素分析[J].现代预防医学,2010,37(8):1420-1422.
291. 王玲,卞茜,张维平, 等.研究生新生自杀态度与影响因素调查[J].上海精神医学,2010,22(2):73-77. DOI:10.3969/j.issn.1002-0829.2010.02.003.
292. 黄辛,蔡篮,檀倩影, 等.医学类高等职业院校心理委员对精神卫生知识知晓率及对精神疾病态度的调查[J].广西医学,2016,38(8):1123-1126,1151. DOI:10.11675/j.issn.0253-4304.2016.08.21.
293. 冯天达,马贞玉,黄慧, 等.医学生心理骨干危机干预知识水平培训效果评价[J].中国健康教育,2013,29(6):545-546,569.
294. 张浩.医学生心理健康与自杀态度分析[J].上海交通大学学报(医学版),2008,28(z1):14-16.
295. 秦英,王志忠,井劲云, 等.医学生与非医学生对精神障碍态度的比较[J].宁夏医科大学学报,2015,37(10):1174-1177. DOI:10.16050/j.cnki.issn1674-6309.2015.10.014.
296. 徐瑜,欧光忠,吴祖达.医学生自杀态度及影响因素调查[J].中国健康教育,2006,22(10):756-759. DOI:10.3969/j.issn.1002-9982.2006.10.010.
297. 程伟彬,杨翌,郜艳晖.医学生自杀态度与社会支持关系[J].中国公共卫生,2008,24(8):938-939.
298. 卢和丽,袁也丰,郭明, 等.医学硕士研究生自杀态度及影响因素分析[J].中国公共卫生,2010,26(11):1381-1383.
299. 郑亚楠,张志花,龚茜.医学研究生精神卫生知识知晓对精神障碍患者歧视影响调查分析[J].中国医学伦理学,2017,30(8):1002-1005,1029. DOI:10.12026/j.issn.1001-8565.2017.08.19.
300. 胡细玉.医院工作人员自杀态度及影响因素分析[J].中国保健营养（下旬刊）,2012,22(11):4824-4825. DOI:10.3969/j.issn.1004-7484(x).2012.11.723.
301. 蒋庆飞,徐秋萍,陈明敏, 等.抑郁症患者的自杀态度、抑郁水平和应对方式的相关性[J].解放军护理杂志,2008,25(7):15-17. DOI:10.3969/j.issn.1008-9993.2008.07.006.
302. 贺春荣,申彦丽,梁执群, 等.抑郁症患者自杀态度与自杀意念的关系研究[J].中国健康心理学杂志,2012,20(4):509-510.
303. 王强,梁海艳,金立元, 等.银川市普通居民精神卫生知识认知情况调查分析[J].宁夏医学杂志,2015,37(4):342-344. DOI:10.13621/j.1001-5949.2015.04.0342.
304. 陈奇,吴金萍,郑猛雷, 等.鄞州区居民心理健康知识知晓情况及影响因素分析[J].预防医学,2018,30(4):374-376,381. DOI:10.19485/j.cnki.issn2096-5087.2018.04.014.
305. 邹敏.影响医学生自杀态度的应激因素分析[J].中国健康心理学杂志,2015,23(11):1720-1723. DOI:10.13342/j.cnki.cjhp.2015.11.033.
306. 王爱青.优质护理干预对精神分裂症患者健康知识知晓程度及服药依从性的影响[J].齐鲁护理杂志,2013,19(19):19-21. DOI:10.3969/j.issn.1006-7256.2013.19.008.
307. 张晓,韦波,唐峥华, 等.邮政快递人员抑郁障碍相关知识及态度的调查[J].中国健康教育,2013,29(2):151-154.
308. 沈春玲,李秋洁,周郁秋, 等.有机磷药物自杀患者心理行为干预的研究[J].护理管理杂志,2008,8(10):15-17. DOI:10.3969/j.issn.1671-315X.2008.10.006.
309. 何承林,黄泽娇,林良平, 等.粤西大学生自杀态度及与社会支持的相关研究[J].湛江师范学院学报,2010,31(1):172-176. DOI:10.3969/j.issn.1006-4702.2010.01.040.
310. 肖垚南,陈妙扬,陈丁玲, 等.云浮市普通人群心理健康知识知晓率及对精神疾病态度调查[J].中国医药导报,2015,(12):75-78.
311. 何祖才,王文伶.在校医学生对精神病知识的了解与态度的调查分析[J].中国民康医学,2016,28(11):57-58. DOI:10.3969/j.issn.1672-0369.2016.11.026.
312. 徐慧兰,马贞玉,肖水源, 等.长沙市大学生自杀预防知识、态度及其影响因素[J].中国心理卫生杂志,2007,21(9):654-658. DOI:10.3321/j.issn:1000-6729.2007.09.020.
313. 李平非,王晓玲,彭元.长沙市老年期精神卫生知识问卷调查[J].中国老年学杂志,2013,33(13):3254-3255. DOI:10.3969/j.issn.1005-9202.2013.13.141.
314. 葛小平,周甲龙.长沙市民精神卫生知识知晓率调查分析[J].神经疾病与精神卫生,2013,13(3):244-247. DOI:10.3969/j.issn.1009-6574.2013.03.008.
315. 王晓玲,李平非,彭元, 等.长沙市中学生心理卫生知识知晓率调查[J].中国健康心理学杂志,2012,20(2):248-250.
316. 穆燕.正念认知疗法干预对抑郁症患者心理状态及自杀意念的影响[J].海军医学杂志,2017,38(6):530-533. DOI:10.3969/j.issn.1009-0754.2017.06.018.
317. 高强,姚明解,阎秀芳, 等.郑州市管城区居民精神卫生知识知晓情况[J].郑州大学学报（医学版）,2014,(6):829-832. DOI:10.13705/j.issn.1671-6825.2014.06.017.
318. 阎琳,刘军,姚明解, 等.郑州市居民对精神疾病患者态度调查[J].中国公共卫生,2015,31(8):987-989. DOI:10.11847/zgggws2015-31-08-03.
319. 姚明解,阎秀芳,常战军, 等.郑州市居民精神卫生知识知晓率调查[J].中国心理卫生杂志,2013,27(9):682-685. DOI:10.3969/j.issn.1000-6729.2013.09.009.
320. 刘国义,高艳华,张艳娥.中等专业学校在校学生的心理状况及自杀态度和意念[J].中国临床康复,2006,10(38):42-44. DOI:10.3321/j.issn:1673-8225.2006.38.018.
321. 赵荣江,牛雅娟,杨少杰, 等.中国北方城市和农村居民自杀态度的调查[J].临床精神医学杂志,2015,(4):234-237.
322. 孙霞,李献云,费立鹏.中国北方两地城乡居民常见精神卫生知识知晓情况现况调查[J].中国心理卫生杂志,2009,23(10):729-733,741. DOI:10.3969/j.issn.1000-6729.2009.10.019.
323. 杨清艳,牛雅娟,杨少杰, 等.中国北方两地居民对常见精神疾病知晓情况的调查[J].四川精神卫生,2014,(6):490-493,494. DOI:10.11886/j.issn.1007-3256.2014.06.003.
324. 王小平,刘铁桥,郝伟, 等.中国部分地区精神科医生对精神障碍者及其处置政策态度的调查[J].国际精神病学杂志,2010,37(1):1-4.
325. 李飞,肖水源,黄志平, 等.中国三城市精神健康素养调查[J].中国心理卫生杂志,2009,23(12):883-887. DOI:10.3969/j.issn.1000-6729.2009.12.013.
326. 王慧莹,杨放如,张杰, 等.中国乡镇医生精神疾病知识以及对精神病人态度的调查[J].中国临床心理学杂志,2018,26(1):105-112. DOI:10.16128/j.cnki.1005-3611.2018.01.024.
327. 王向阳,任衍镇,李元春, 等.中山市中小学教师精神卫生知识知晓情况调查分析[J].四川精神卫生,2015,(3):248-251. DOI:10.11886/j.issn.1007=3256.2015.03.018.
328. 陈茂卉,周建国,王宏刚, 等.中学生对自杀态度的多维度分析[J].健康心理学杂志,2003,11(5):394-396. DOI:10.3969/j.issn.1005-1252.2003.05.036.
329. 易静,李桂蓉,蒋迎春, 等.中学生物质滥用及艾滋病相关知识和行为分析[J].现代预防医学,2003,30(4):478-480. DOI:10.3969/j.issn.1003-8507.2003.04.008.
330. 杨阳,潘运,薛应翠, 等.中专学生人际信任与自杀态度的相关研究[J].保健医学研究与实践,2016,13(2):11-13,21. DOI:10.11986/j.issn.1673-873X.2016.02.003.
331. 刘彩谊,张惠敏,李焕, 等.重度网络游戏成瘾男性青少年的自杀态度与家庭影响因素[J].中国药物依赖性杂志,2013,22(1):56-60. DOI:10.3969/j.issn.1007-9718.2013.01.014.
332. 王丽琴,陈燕,钱春芳.重固镇居民精神卫生健康教育效果评价[J].上海医药,2012,(24):39-40. DOI:10.3969/j.issn.1006-1533.2012.24.018.
333. 赵永萍,韦敏,梅明秋, 等.重庆市中小学生孤独情绪体验和自杀态度的特点与关系[J].中国健康心理学杂志,2017,25(1):117-121. DOI:10.13342/j.cnki.cjhp.2017.01.028.
334. 吕臻,艾明,况利, 等.重庆市自杀未遂大学生的自杀态度和社会支持系统的调查[J].重庆医学,2014,(26):3474-3477. DOI:10.3969/j.issn.1671-8348.2014.26.023.
335. 张勇,田梅,白珍, 等.重性精神疾病管理治疗项目保定市示范区精神卫生知识调查[J].中国健康心理学杂志,2011,19(1):1-3.
336. 杨玲,樊召锋.自杀态度与自杀倾向的相关分析——大学生自杀风险评估与危机干预研究[J].甘肃社会科学,2009,(1):235-237. DOI:10.3969/j.issn.1003-3637.2009.01.059.
337. 徐宝昌,何为民,甘建光.自杀姿势的分离性障碍患者自杀态度研究[J].中国全科医学,2004,7(15):1060-1061. DOI:10.3969/j.issn.1007-9572.2004.15.020.
338. 姜杨,黄金文,徐瑾.综合性医院护士抑郁症知识知晓率的调查[J].护理与康复,2009,8(7):558-559. DOI:10.3969/j.issn.1671-9875.2009.07.006.
339. 吴秋霞,陈淑宝,罗小阳, 等.综合医院非精神科医师对广泛性焦虑障碍的知晓情况调查[J].中国临床心理学杂志,2016,24(5):894-899. DOI:10.16128/j.cnki.1005-3611.2016.05.028.
340. 熊宇,胡德英,刘义兰, 等.综合医院护士对住院患者自杀预防认知的调查研究[J].护理学杂志,2016,31(1):59-62. DOI:10.3870/j.issn.1001-4152.2016.01.059.
341. 潘红英,施建英,吴丽仙, 等.综合医院护士精神病知识短期培训效果分析[J].护理学报,2008,15(6):44-45. DOI:10.3969/j.issn.1008-9969.2008.06.016.
342. 赵永忠,何金彩,董斐蓉, 等.综合医院基于网络的精神卫生知识培训效果评价[J].中华健康管理学杂志,2014,8(4):226-228. DOI:10.3760/cma.j.issn.1674-0815.2014.04.004.
343. 李丽娜,高凌云,曾宏翔, 等.综合医院医护人员对抑郁症认知调查[J].中国健康心理学杂志,2015,23(11):1634-1636. DOI:10.13342/j.cnki.cjhp.2015.11.011.
344. 李杰.罪犯自杀态度及其相关影响因素[J].中国健康心理学杂志,2006,14(3):334-335. DOI:10.3969/j.issn.1005-1252.2006.03.040.
345. 胡宓,肖水源.汶川地震受灾地区灾后社区、乡镇卫生院工作者精神卫生服务能力评估[J].中国心理卫生杂志,2012,26(2):98-103. DOI:10.3969/j.issn.1000-6729.2012.02.008.
346. 黎泽明,黎燕宁,韦波, 等.城市社区与农村乡镇基层精神卫生工作人员专业能力比较研究[J].现代预防医学,2017,44(14):2545-2548.
347. 李献云,费立鹏,杨少杰, 等.城乡居民对自杀的接受程度及其与自杀意念和行为的关系[J].中国心理卫生杂志,2009,23(10):734-739. DOI:10.3969/j.issn.1000-6729.2009.10.012.
348. 徐小明,况利,艾明, 等.重庆市大学生对自杀态度的调查[J].中华流行病学杂志,2013,34(6):569-572. DOI:10.3760/cma.j.issn.0254-6450.2013.06.007.
349. 袁水莲,李伟红.健康教育沙龙对精神分裂症病人疾病知识掌握情况及服药依从性的影响[J].护理研究,2014,(17):2121-2122. DOI:10.3969/j.issn.10096493.2014.17.036.
350. 李会,穆喜术,李淑芬, 等.综合护理干预措施对抑郁症患者自杀态度和自我接纳的影响[J].神经疾病与精神卫生,2016,16(5):574-577. DOI:10.3969/j.issn.1009-6574.2016.05.023.
